# Recessive, gain-of-function toxicity in an APOL1 BAC transgenic mouse model mirrors human APOL1 kidney disease

**DOI:** 10.1242/dmm.048952

**Published:** 2021-08-05

**Authors:** Gizelle M. McCarthy, Angelo Blasio, Olivia G. Donovan, Lena B. Schaller, Althea Bock-Hughes, Jose M. Magraner, Jung Hee Suh, Calum F. Tattersfield, Isaac E. Stillman, Shrijal S. Shah, Zsuzsanna K. Zsengeller, Balajikarthick Subramanian, David J. Friedman, Martin R. Pollak

**Affiliations:** 1 Nephrology Division, Department of Medicine, Beth Israel Deaconess Medical Center and Harvard Medical School, Boston, MA 02215, USA; 2 Dept. of Pathology, Beth Israel Deaconess Medical Center and Harvard Medical School, Boston, MA 02215, USA

**Keywords:** APOL1, Kidney, Mouse, Transgenic

## Abstract

People of recent sub-Saharan African ancestry develop kidney failure much more frequently than other groups. A large fraction of this disparity is due to two coding sequence variants in the *APOL1* gene. Inheriting two copies of these *APOL1* risk variants, known as G1 and G2, causes high rates of focal segmental glomerulosclerosis (FSGS), HIV-associated nephropathy and hypertension-associated end-stage kidney disease. Disease risk follows a recessive mode of inheritance, which is puzzling given the considerable data that G1 and G2 are toxic gain-of-function variants. We developed coisogenic bacterial artificial chromosome (BAC) transgenic mice harboring either the wild-type (G0), G1 or G2 forms of human *APOL1*. Expression of interferon gamma (IFN-γ) via plasmid tail vein injection results in upregulation of APOL1 protein levels together with robust induction of heavy proteinuria and glomerulosclerosis in G1/G1 and G2/G2 but not G0/G0 mice. The disease phenotype was greater in G2/G2 mice. Neither heterozygous (G1/G0 or G2/G0) risk variant mice nor hemizygous (G1/−, G2/−) mice had significant kidney injury in response to IFN-γ, although the heterozygous mice had a greater proteinuric response than the hemizygous mice, suggesting that the lack of significant disease in humans heterozygous for G1 or G2 is not due to G0 rescue of G1 or G2 toxicity. Studies using additional mice (multicopy G2 and a non-isogenic G0 mouse) supported the notion that disease is largely a function of the level of risk variant APOL1 expression. Together, these findings shed light on the recessive nature of APOL1-nephropathy and present an important model for future studies.

## Abstract (precis)

**Summary:** The APOL1 transgenic mouse model develops interferon-induced variant-dependent kidney disease that recapitulates human recessive gain of function phenotype.

## INTRODUCTION

Individuals of recent African ancestry have an increased risk of developing non-diabetic kidney disease. Much of this increased risk is attributable to two variants in the apolipoprotein L1 (*APOL1*) gene that originated in sub-Saharan Africa (Friedman et al., 2011; Genovese et al., 2010; Kopp et al., 2011; Tzur et al., 2010). These variants, called G1 and G2, increase the risk of several forms of kidney disease under a recessive model of inheritance. The common alleles of *APOL1*, referred to as G0, confer humans with resistance to trypanosome *Trypanosoma brucei* infection through ion channel formation and trypanolysis but do not protect against the two subspecies that cause human disease: *T. b. rhodesiense* and *T. b. gambiense* (Molina-Portela et al., 2005; Pérez-Morga; et al., 2005). G1, defined by the substitution of two amino acids (aa), i.e. S342G (locus accession: rs73885319) and I384M (locus accession: rs60910145), and G2, defined by dual deletion of aa N388 and Y389 (del388N389Y; locus accession: rs71785313) appear to have arisen in sub-Saharan Africa, and rose quickly to high frequency because they provided a selective advantage in regions with pathogenic trypanosomes (Friedman et al., 2011; Genovese et al., 2010; Thomson et al., 2014; Cooper et al., 2017). Whereas inheriting a single copy of the G1 or G2 *APOL1* alleles is sufficient to enhance protection against resistant trypanosomes, inheriting two copies (one from each parent) substantially increases the risk of developing kidney disease.

APOL1-nephropathy risk follows a recessive mode of inheritance. In contrast to most recessive diseases, an increasing body of evidence suggests that the G1 and G2 alleles lead to toxic gain-of-function changes of the APOL1 protein. APOL1 is not required for normal kidney function, as evidenced by the fact that only genomes of higher-order primates encode a functional APOL1 protein; moreover, at least one human has been identified who is completely null for *APOL1* while having no apparent kidney dysfunction (Johnstone et al., 2012; Smith and Malik, 2009). Furthermore, cell and animal models show that exogenous *APOL1* expression causes cytotoxicity. However, recessive gain of function mutations are rare and, although it has been speculated that the G0 allele is protective in the heterozygous state, to date there is limited evidence to support this idea (Limou et al., 2015).

APOL1-nephropathy has incomplete penetrance, with an estimated 15% lifetime risk for those with a high-risk genotype developing end-stage kidney disease (ESKD), suggesting there are non-genetic factors that modify risk (Dummer et al., 2015). The odds ratio (OR) also varies depending on the type of kidney disease, with an OR of ∼17 for focal segmental glomerulosclerosis (FSGS), ∼29-89 for HIV-associated nephropathy (HIVAN) but no increased risk of incident diabetic kidney disease. Many forms of APOL1-nephropathy involve viral infections or an inflammatory trigger, suggesting that immune mediators may function as a ‘second hit’ (Kasembeli et al., 2015; Larsen et al., 2013). Supporting this notion, treatment with interferon leads to increased APOL1 expression *in vitro*, and has resulted in collapsing nephropathy in people with high-risk genotypes (Nichols et al., 2015; Markowitz et al., 2010). Recently, collapsing nephropathy has been observed in individuals with SARS-CoV-2 infection and high-risk *APOL1* genotypes (Magoon et al., 2020; Sharma et al., 2020; Wu et al., 2020). Although studies have determined that interferon upregulates *APOL1* gene expression through binding of interferon-responsive elements, it remains unclear how this increased expression drives the development of kidney disease (Nichols et al., 2015; Beckerman et al., 2017; Friedman and Pollak, 2020).

Thus, the mechanisms underlying APOL1-nephropathy remain unclear. *In vivo* studies are difficult because *APOL1* is not present in conventional model systems. Several transgenic mouse models have been developed to study APOL1-nephropathy, but none of these models develop both the proteinuria and the histologic lesions observed in humans in response to a known trigger (Beckerman et al., 2017; Bruggeman et al., 2016; Okamoto et al., 2018; Kang et al., 2018; Aghajan et al., 2019; Kumar et al., 2018). To provide further insight into the genetics of APOL1-nephropathy, we developed a set of congenic bacterial artificial chromosome (BAC) transgenic mouse models. These transgenic animals carry the full human *APOL1* and flanking regulatory sequences at the same insertion site to test the consequences of physiological expression of APOL1.

We used interferon plasmid injection to increase expression of the G0 and risk variant forms of APOL1 in our human *APOL1*-expressing transgenic mice. We quantified APOL1 protein levels in kidney, characterized mice that are either hemizygous or homozygous for the different *APOL1* alleles, assessed the kidney disease phenotype of mice with different *APOL1* genotypes and cross-bred mice to evaluate the concept of ‘G0 rescue’ on risk variant toxicity. Our findings demonstrate that interferon-driven expression of untagged APOL1 protein from genomic DNA recapitulates the genetics and disease phenotype observed in humans. The similarities between APOL1-nephropathy in this mouse model and humans present an opportunity to both understand disease mechanisms and to test potential APOL1-targeted therapies.

## RESULTS

### Generation and characterization of transgenic mouse lines

**Fig. 1.**
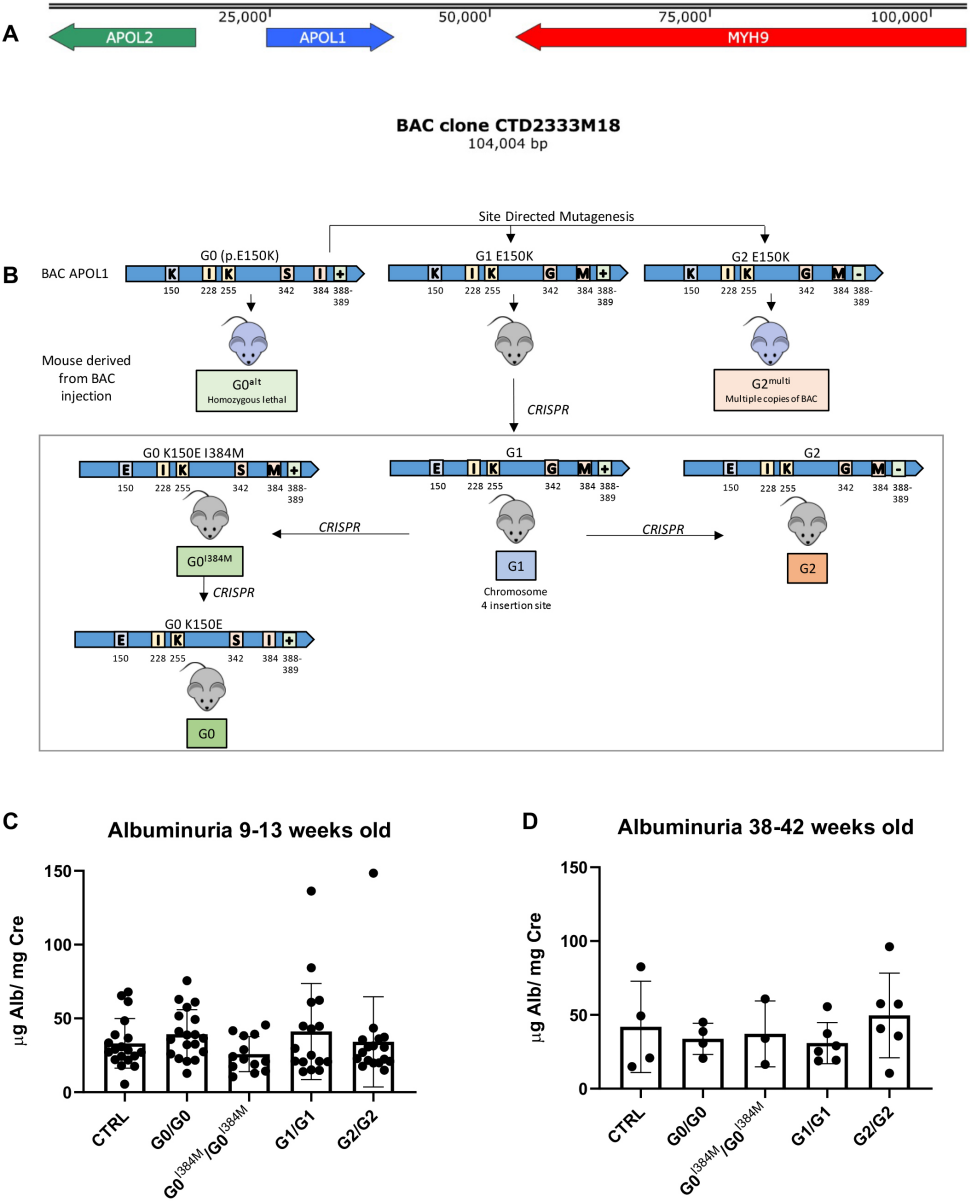
***APOL1* transgenic mouse development and baseline proteinuria.** (A) *APOL1* transgenic mice were created using a 104 kb BAC that contains *APOL1* (G0) and parts of *APOL2* (exons 1-5) and *MYH9* (exons 4-41). (B) *APOL1* transgenic mice were originally created using a BAC that contains *APOL1* (p.E150K haplotype). This BAC contained G0 *APOL1*; G1 and G2 variants were generated using site-directed mutagenesis. The BACs were injected into FVB/NJ oocytes resulting in three mouse lines, i.e. G0^alt^, G1^E150K^ and G2^multi^. Of these, the G0^alt^ mice were unable to breed homozygous and G2^multi^ had multiple copies of the BAC. These two lines were used only used to address questions regarding APOL1 amounts and G0 rescue. The third line, G1, was further mutated using CRISPR, ultimately generating four additional lines: G0^I384M^ and its follow-up line G0, as well as the G1 and G2 lines, all of which have a single copy of *APOL1* integrated into the same chromosome 4 locus. These four mouse lines were used to assess differences between the *APOL1* genotypes. Amino acids at the variable sites are shown for each mouse and the names used for each mouse line are indicated in the colored boxes. (C/D) Quantification of albuminuria in control and transgenic mice at 9-13 (C) and 38-42 weeks of age (D). Shown is the means±s.d. of individual data. No significant differences were found (one-way ANOVA with Tukey's multiple comparisons).

*APOL1* transgenic mice were created using a 104 kb BAC that contains the human *APOL1* gene (14.5 kB) and surrounding genomic DNA (Fig. 1A,B). By using CRISPR mutagenesis, we created four congenic *APOL1* mouse lines, i.e. G0, G0^I384M^, G1 and G2, from a single founder line. Given recent evidence regarding the importance of the *APOL1* sequence in regions outside the sequence that defines the G1/G2 alleles, transgenic mice were generated so that the coding sequence of the entire allele-associated haplotype was identical to that in humans (Lannon et al., 2019). The aa sequence for G0 mice included E150, I228, and K255, which represent the human p.M228I G0 haplotype (Thomson et al., 2014). The G1 and G2 mice contained E150, which is consistent with the naturally occurring sequence of the human haplotype that contains APOL1 variants G1 and G2 (Thomson et al., 2014).

In humans, APOL1 is present at high levels in the plasma, and at lesser amounts in the liver and kidney (Ma et al., 2015; Monajemi et al., 2002; Page et al., 2001; Shukha et al., 2017). To confirm that protein distribution in tissues of these transgenic mice is similar to that humans, we compared APOL1 levels in plasma, liver, and kidney. Western blotting revealed that, in these transgenic mice, APOL1 protein levels are high in the plasma, levels in the liver are low and least in the kidney (Fig. S2A). Comparison of plasma from APOL1 transgenic mice with that from humans with different *APOL1* genotypes confirmed similar APOL1 protein levels (Fig. S2B).

To determine whether expression of a human *APOL1* gene risk variant caused any kidney disease, the urinary albumin to creatinine ratio was measured in control (FVB/NJ) and transgenic mice. No differences were seen between any of these mice (Fig. 1B), even at 9 months of age (Fig. 1C). Similarly, no evidence of kidney damage or kidney abnormalities was seen by light microscopy in mice of any homozygous *APOL1* genotype (Fig. S3). Therefore, transgenic expression of human APOL1 in mice does not seem to cause any apparent kidney disease, irrespective of *APOL1* genotype. This finding is consistent with other APOL1 mouse models and the fact that most humans with a high-risk *APOL1* genotype do not develop kidney disease (Aghajan et al., 2019; Dummer et al., 2015; Ryu et al., 2019).

### The INF-γ-expressing pCpGfree plasmid induces prolonged expression of glomerular APOL1

Not all humans with two copies of *APOL1* risk variants develop kidney disease, suggesting that some other factor or factors (second hit) must also be present to cause kidney damage. For people with a high-risk genotype, there is a strong association between HIV infection and the development of kidney disease (Kopp et al., 2011). Previous reports indicate that interferon gamma (IFN-γ), which is produced in response to HIV infection, is a potent inducer of APOL1 expression *in vitro* and that IFN-γ treatment can cause collapsing focal segmental glomerulosclerosis (FSGS) in humans with a high-risk *APOL1* genotype (Nichols et al., 2015). However, the half-life of interferon is very short *in vivo*, making it difficult to model the sustained levels observed during human HIV infection or IFN-γ treatment in mice (Roff et al., 2013; Watanabe et al., 2019; Markowitz et al., 2010). This issue can be circumvented by utilizing an IFN-γ-expressing pCpGfree plasmid that lacks CpG motifs (pCpG-Muγ), expression of which results in sustained serum IFN-γ levels for weeks after tail vein injection (Mitsui et al., 2009). Elimination of most CpG sequences reduces the inflammatory effects of bacterial double-stranded DNA.

**Fig. 2.**
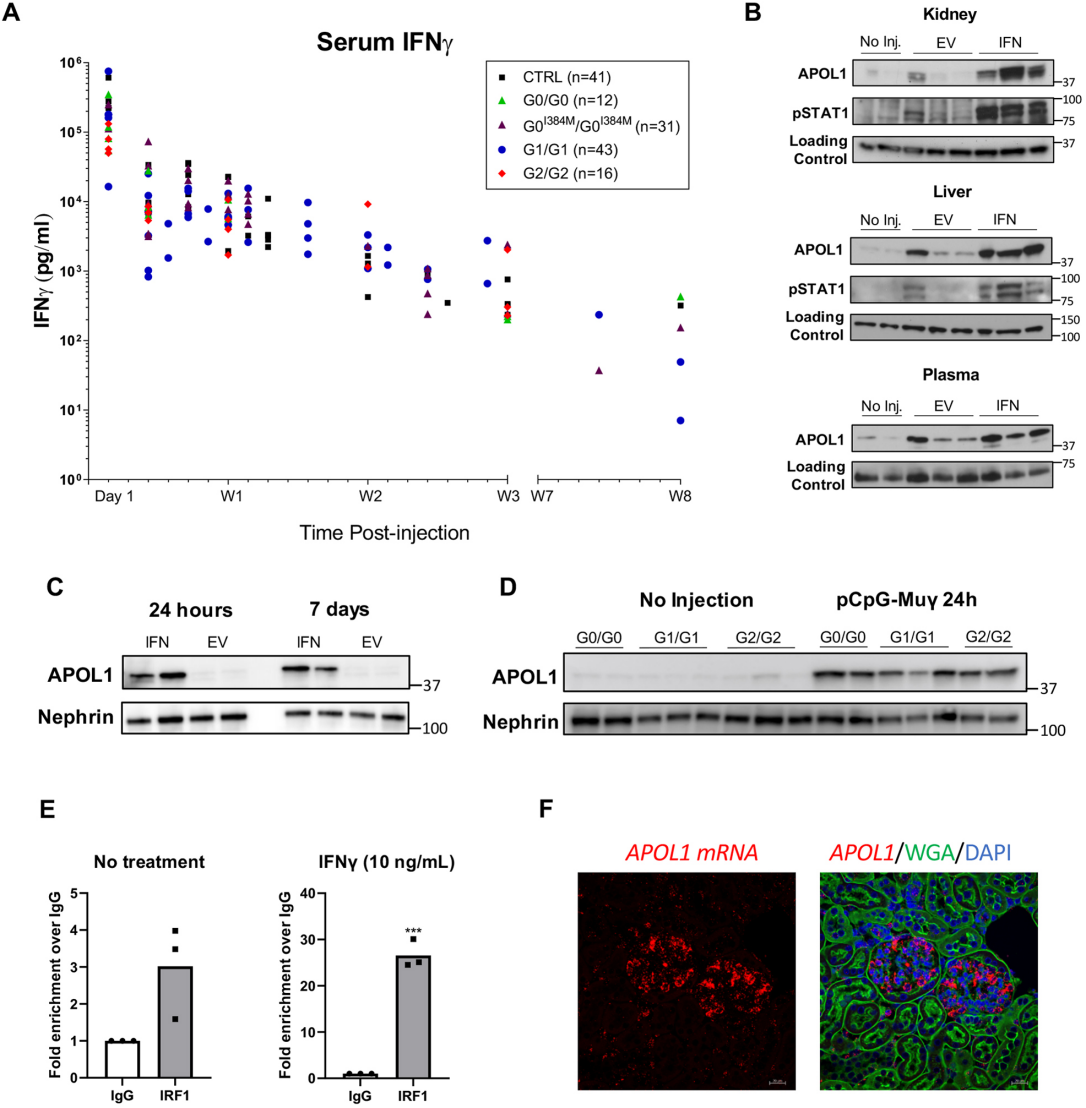
**Injection of pCpG-Muγ induces expression of APOL1 in glomeruli.** Mice were hydrodynamically injected with 0.3 µg of pCpG-Muγ (IFN-γ) for sustained levels of IFN-γ or with 0.3 µg pCpG-EV (EV) as control. (A) Serum IFN-γ levels measured using ELISA following injection of pCpG-Muγ. Individual data points for all four transgenic genotypes plus non-transgenic FVB controls (CTRL) are shown, *n* numbers are shown in the figure legend. IFN-γ levels before-injection (day 0) were undetectable (not shown). (B) Western blots, showing APOL1 levels (Abcam) in G0^I384M^ kidney, liver and plasma 24 h after injection of EV (*n*=3), IFN-γ (*n*=3), or without injection (No Inj., *n*=2). Loading controls were GAPDH (kidney), vinculin (liver) and transferrin (plasma). (C) Levels of APOL1 (Sigma-Aldrich) in glomeruli of G1 mice 24 h and 7 days after injection of pCpG-Muγ (IFN) or pCpG-EV (EV); *n*=2. (D) Levels of glomerular APOL1 (Sigma-Aldrich) at baseline and 24-h after pCpG-Muγ injection (G0/G0 here is G0^I384M^/G0^I384M^); *n*=2 for G0/G0, *n*=3 for G1/G1 and G2/G2). In C and D, nephrin is shown as a control for glomerular preparation. (E) Chromatin immunoprecipitation was performed on podocytes derived from G0/G0 mice using antibody against IRF1 (D5E4, Cell Signaling Technology) or Rabbit IgG (2729S, Cell signaling Technology). Fold-enrichment over IgG is shown at baseline or at 6 h after treatment with 10 ng/ml IFNγ. *n*=3, ***-*P*<0.0005, Student's *t*-test. (F) *In situ* hybridization of *APOL1* in kidney of G1/G1 mice 24-h after pCpG-Muγ injection. Images of FVB control and non-injected G1/G1 mice are shown in Fig. S7.

In this *APOL1*-transgenic model, hydrodynamic tail vein injection of 0.3 µg of pCpG-Muγ produced high serum IFN-γ levels that remained elevated above baseline (undectable; lower limit of assay was 16 pg/ml) levels for at least 8 weeks (Fig. 2A). STAT1, which is phosphorylated in response to interferon (pSTAT1) and acts as a transcription factor for *APOL1*, was used to assess the IFN-γ response in different cell and tissue types (Beckerman et al., 2017;Reich, 2007). In response to pCpG-Muγ, protein levels of pSTAT1 and APOL1 were increased in the kidney cortex and, to a lesser extent, in the liver and plasma (Fig. 2B). Hydrodynamic injection of control plasmid pCpG-EV (the pCpGfree plasmid without insert) did not increase APOL1 levels in the kidney, although it somewhat increased levels in the liver, which sustains damage as a result of the hydrodynamic injection itself (Suda and Liu, 2015). Within the kidney, APOL1 was increased in glomeruli of the transgenic mice, where it was still elevated 1 week later (Fig. 2C). Levels of glomerular APOL1 24 h after injection were similar across the different transgenic lines (Fig. 2D). Chromatin immunoprecipitation (ChIP) confirmed that the mouse transcription factor IRF1, which is upregulated in response to IFN-γ, can bind the human *APOL1* promoter (Fig. 2E,Fig. S4).

**Fig. 3.**
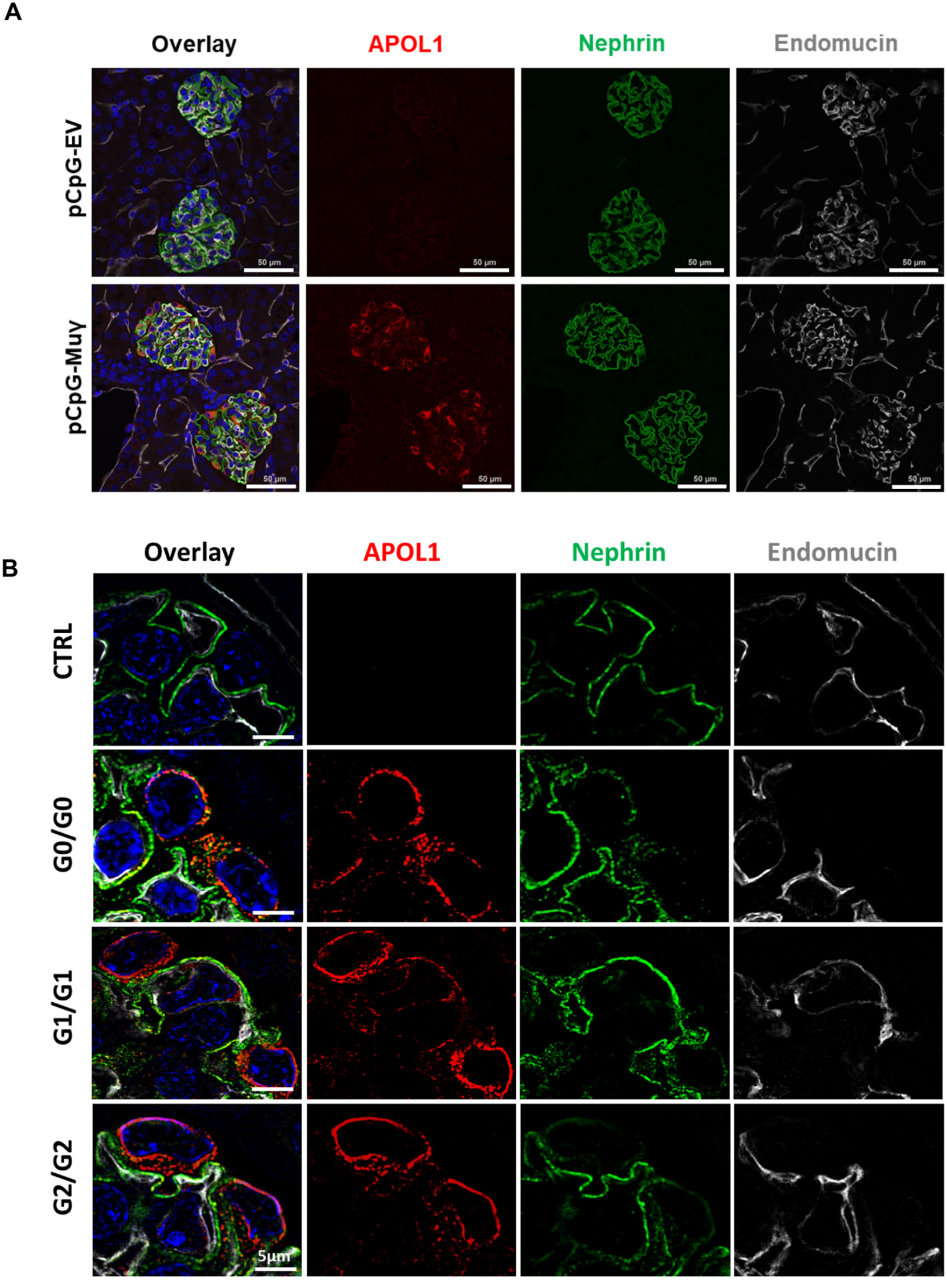
**APOL1 expression is induced in podocytes following pCpG-Muγ injection.** (A) APOL1 expression 1 day after hydrodynamic injection of pCpG-EV or pCpG-Muγ in G1 mice. Kidney tissue was co-stained for APOL1 (Abcam), the podocyte slit diaphragm marker nephrin and the endothelial cell marker endomucin. Scale bars: 50 μM. (B) SIM images of CTRL, G0/G0, G1/G1, and G2/G2 glomeruli 1 day after injection with pCpG-Muγ, co-stained with APOL1 (Abcam), nephrin, and endomucin. Scale bars: 5 μM.

Previous studies suggested that, in human kidney, APOL1 is expressed in different cells, including podocytes, endothelial cells and tubules, although some discrepancies have been attributed to the use of different antibodies against APOL1 (Ma et al., 2015; Madhavan et al., 2011; Scales et al., 2020; Uhlén et al., 2015). To determine APOL1 localization in our mouse model, we co-stained for APOL1, the podocyte slit diaphragm marker nephrin, and the endothelial cell marker endomucin. Immunofluorescence revealed that injection of IFN-γ-expressing vector but not of empty vector, induced APOL1 expression almost exclusively within glomeruli, specifically in podocytes (Fig. 3A). Structured illumination microscopy (SIM) confirmed that, in all genotypes, APOL1 was induced primarily in podocytes, with endothelial cells showing weaker staining (Fig. 3B). Expression within the podocyte was mostly cytoplasmic and colocalized with the ER marker calnexin (Fig. S6); we observed no apparent colocalization within the podocyte foot processes. Additional staining for APOL1 using a different antibody (Proteintech), as well as staining for Wilms tumor protein (WT1) as a nuclear podocyte marker and platelet-derived growth factor receptor beta (PDGFRb) as a mesangial cell marker, confirmed that APOL1 is primarily expressed in podocytes after injection of pCpG-Muγ (Fig. S5A,B). Podocyte localization was also confirmed by using a third APOL1 antibody (Sigma-Aldrich, Fig. S5C). *In situ* hybridization revealed that APOL1 is being produced in glomeruli as opposed to being taken up from freely circulating APOL1 (Fig. 2F, Fig. S7).

### Injection of pCpG-Muγ causes albuminuria in G1/G1 and G2/G2 but not G0/G0 mice

**Fig. 4.**
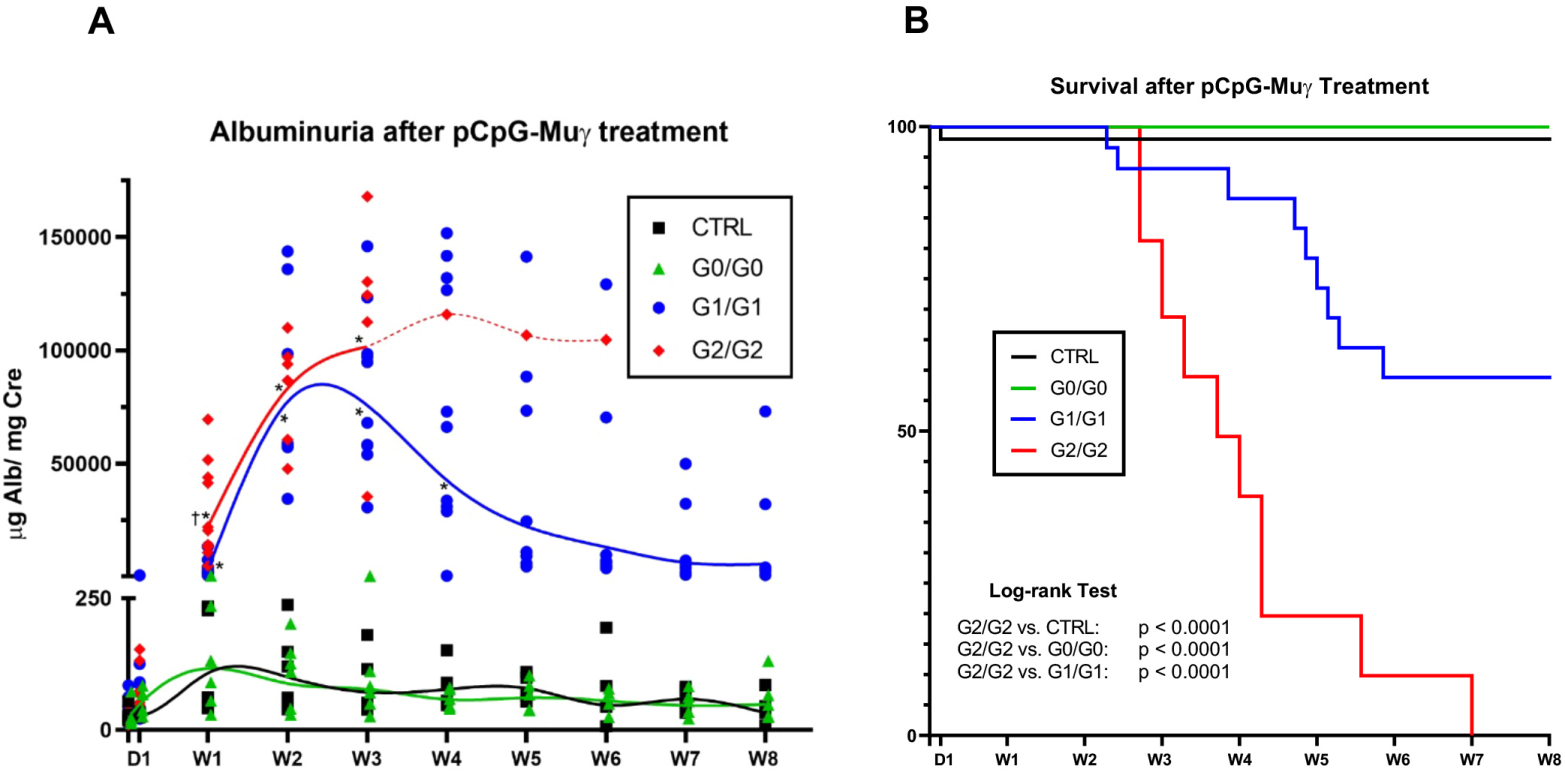
**G1 and G2 mice develop albuminuria and die following pCpG-Muγ injection.** (A) Albuminuria [levels of albumin (Alb) to creatinine (Cre)] was measured up to 8 weeks in response to injection of pCpG-Muy yielding sustained levels of IFN-γ. Individual data points from several experiments are shown and splines were drawn to visualize data trends. The G2 spline converts from a solid to a dashed line at week 3 (W3), with only data from one surviving mouse plotted. * indicate data significantly different from CTRL, G0/G0, and G0^I384M^/G0^I384M^, † indicate data significantly different from G1/G1; *P*<0.05; Mixed effects model with Tukey's multiple comparisons test. (B) Survival curves up to 8 weeks post injection, including mice from all experiments. Individual curves were compared using a Log-rank test; *P*-values passing a Bonferroni corrected threshold of 0.005 were considered significant and are shown. D, day post injection; CTRL, FVB control mice. No significant difference regarding albuminuria or survival was observed between G0/G0 and G0^I384M^/G0^I384M^ (data not shown).

Individuals with high-risk genotypes are more likely to develop albuminuria, and many APOL1-associated nephropathies (e.g. HIVAN and FSGS) often present with nephrotic range proteinuria (Chen et al., 2017; Markowitz et al., 2010; Reidy and Kaskel, 2007; Wyatt et al., 2008). To determine whether IFN-γ treatment can induce genotype-dependent proteinuria, control and *APOL1-*transgenic mice were injected with 0.3 µg of pCpG-Muγ. Urine albumin was measured weekly for 8 weeks (Fig. 4A). Albuminuria (the albumin to creatinine ratio) for the two G0/G0 lines was not significantly different from FVB control mice at any time point. G1/G1 and G2/G2 mice, however, showed a significant increase (∼1000-fold) in albuminuria compared to control or G0/G0 lines at weeks 1, 2 and 3. G1/G1 mice reached peak proteinuria 2-3 weeks post injection, after which levels declined, but never returned to baseline. In contrast, injection of G1/G1 mice with the pCpG-EV control plasmid did not produce any increase in urinary albumin (Fig. S8). G2/G2 mice achieved very high levels of albuminuria at week 3, at which time most of the mice had died (Fig. 4A,B). G2/G2 mice exhibited a trend towards increased albuminuria compared with G1/G1 mice, with a significant difference at week 1 (*P*=0.032), suggesting differences in the biological behavior between the two risk variants. No difference in urine albumin was observed regarding sex in any of the genotypes (Fig. S9). Overall, these data show that *APOL1* G1/G1 and G2/G2 transgenic mice develop severe albuminuria in response to sustained IFN-γ exposure, whereas G0/G0 and non-transgenic mice do not.

### Risk variant mice die of apparent kidney dysfunction

Patients with high-risk *APOL1* genotypes are not only more likely to develop CKD, but also more likely to progress to end-stage kidney disease (ESKD) (Foster et al., 2013; Fine et al., 2012; Kopp et al., 2011). After treatment with pCpG-Muγ, many risk-variant mice died prematurely or required euthanasia, whereas all G0/G0 mice survived (Fig. 4B). Every G2/G2 mouse that had not been euthanized at an experimental endpoint was dead by day 49, with a median survival of 26 days. The death rate for G1/G1 mice was lower than that of G2/G2 mice, with 58% survival 8 weeks post injection and a median life span of 34 days. Comparison between G1 surviving mice and those that died revealed that proteinuria was increased 4 weeks after injection in those mice that later died (Fig. S10), suggesting that death was a consequence of kidney damage.

**Fig. 5.**
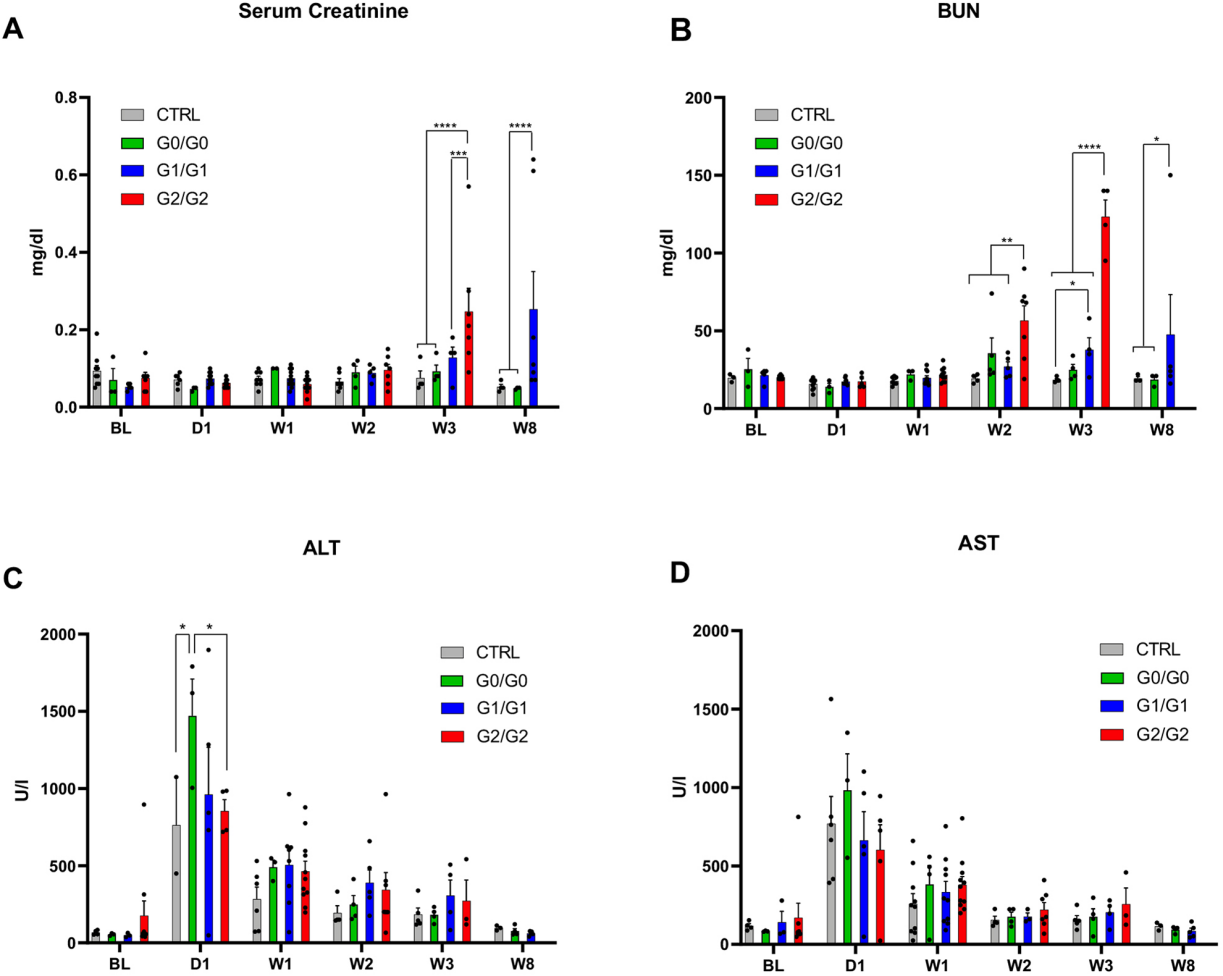
**Markers of kidney dysfunction are elevated in G1 and G2 mice.** (A-D) Measurements of serum creatinine, blood urea nitrogen (BUN), aspartate aminotransferase (AST) and alanine aminotransferase (ALT) (A,B,C and D, respectively) in FVB control (CTRL), G0/G0, G1/G1, and G2/G2 mice. Plotted is the mean+s.e.m. with individual data points shown as dots at day 1 (D1) and up to week (W) 8 post injection. BL, baseline (no IFN-γ injection). ****P<0.0001, ***P<0.001, **P<0.01, *P<0.05, two-way ANOVA with Tukey's multiple comparisons test. G0^I384M^/G0^I384M^ was not significantly different from G0/G0 (data not shown). For the BUN assay two G2/G2 samples were measured at W3 to be >140 mg/dl (i.e. the upper limit of the assay) and are shown as 140 mg/dl.

To determine whether high-risk *APOL1* mice were experiencing kidney failure, we measured markers of kidney and liver function. Levels of serum creatinine and BUN (blood urea nitrogen), which increase when the kidneys are not filtering effectively, were elevated in G2/G2 mice 3 weeks post injection (Fig. 5A,B). G1/G1 mice, which died later and at a lesser rate, had elevated serum creatinine levels 8 weeks post injection. To rule out the possibility that the mice were dying of liver failure, aspartate aminotransferase (AST) and alanine aminotransferase (ALT) were also measured (Fig. 5C,D). Both enzymes increased on day 1 in all genotypes, consistent with liver injury caused by the hydrodynamic injection; it then declined over time with no significant genotypic differences. Additional necropsy analysis of G2/G2 mice found no apparent evidence of damage in any other organs that could explain the high rate of mortality in these mice (data not shown). Overall, these results suggest that high-risk *APOL1* mice died due to kidney failure or from sequelae of extreme proteinuria.

### G1 and G2 mice develop FSGS-like disease

**Fig. 6.**
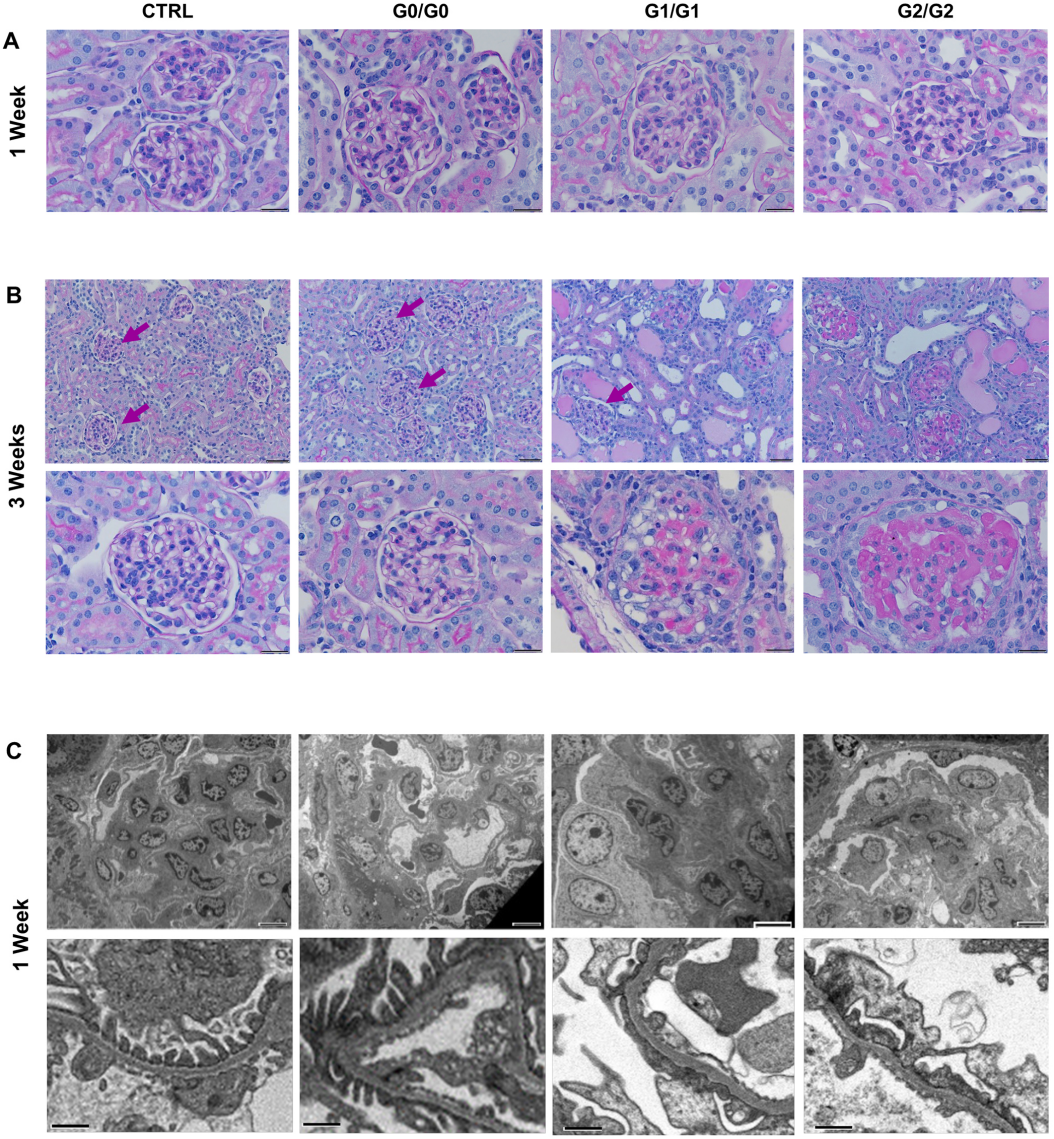
**G1 and G2 mice develop foot process effacement and glomerulosclerosis.** (A,B) Representative images of PAS-stained sections of the kidney cortex at 1 week (A) and 3 weeks (B) after pCpG-Muγ injection. No pathology was observed 1 week after injection, but sclerotic glomeruli (black arrows) were found in G1/G1 and G2/G2 mice 3 weeks post injection. Purple arrows mark normal glomeruli. Bottom images in B show magnified glomeruli as indicated in A. Scale bars: 10 µM (A, B bottom), 20 µM (B top). (C) TEM images of kidney cortex sections at 1 week post injection show foot process effacement in G1/G1 and G2/G2 mice. Scale bars: 6 µM (top) and 1 µM (bottom).

In humans with APOL1-nephropathy, tissue biopsy analysis often reveals glomerulosclerosis (Kopp et al., 2015; Fine et al., 2012; Markowitz et al., 2010). Histopathological analysis of the IFN-γ-treated *APOL1*-transgenic mice revealed that G1/G1 and G2/G2 mice also developed glomerulosclerosis, with characteristics similar to those seen in humans. One week post injection all genotypes appeared mostly normal by light microscopy (Fig. 6A) but simplification of the interdigitating podocyte foot processes, often referred to as foot process effacement commonly seen in FSGS, was visible in G1/G1 and G2/G2 mice by using electron microscopy (Fig. 6C) (Taneda et al., 2015). By 3 weeks post injection both G1/G1 and G2/G2 mice had developed severe glomerulosclerosis, with some features of collapsing glomerulopathy (Fig. 6B). Control and G0/G0 mice appeared normal at all time-points. No differences were observed between G0/G0 and G0^I384M^/G0^I384M^ kidneys, confirming that the S342G substitution is required for G1-mediated toxicity in this mouse system (Fig. S11A). Together, these results show that transgenic expression of the human *APOL1* gene under control of its endogenous promotor can recapitulate important features of APOL1-nephropathy seen in humans.

### G1 and G2 mice show evidence of podocyte damage and loss over the course of the disease

**Fig. 7.**
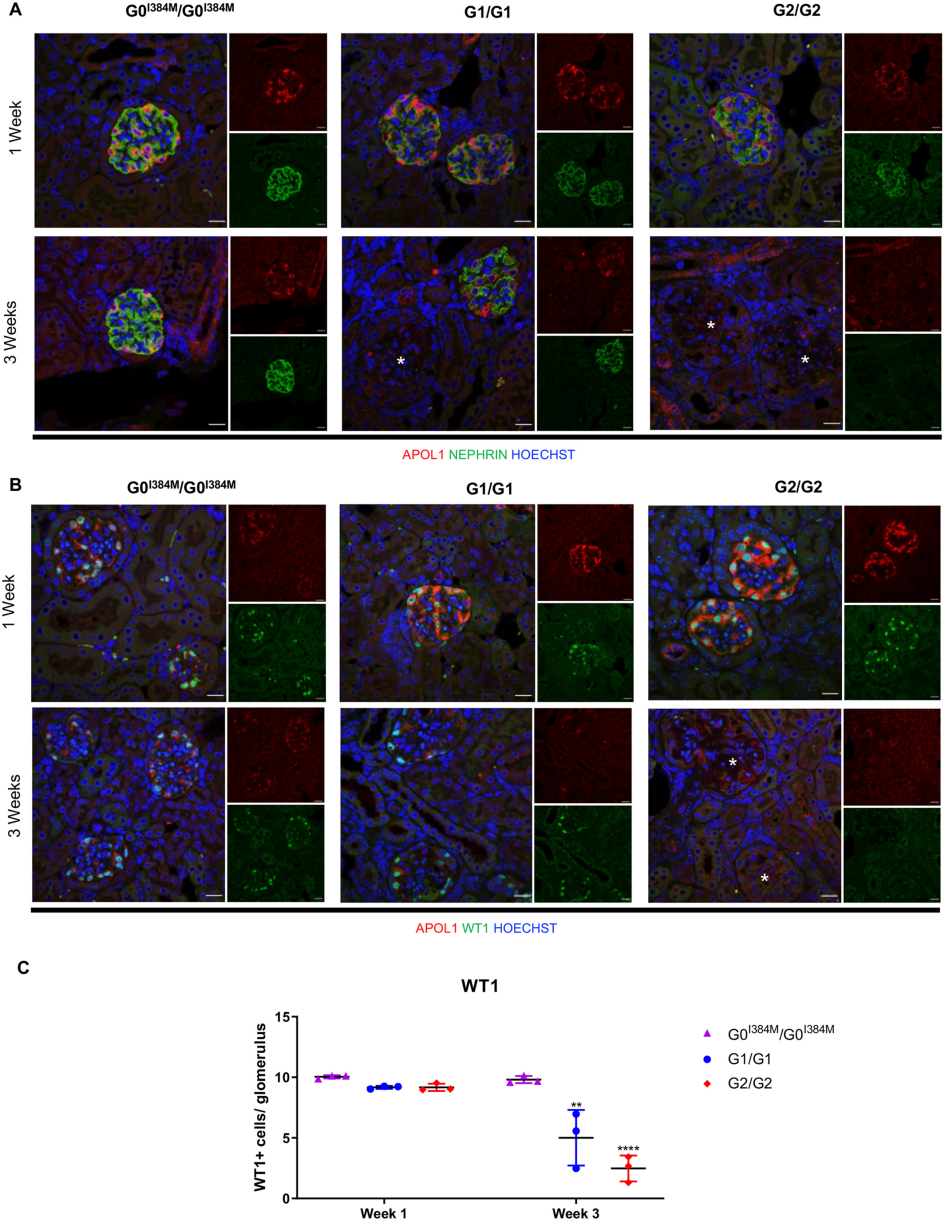
**G1 and G2 mice show evidence of podocyte loss.** G0^I384M^/G0^I384M^, G1/G1 and G2/G2 mice were injected with pCpG-Muy for sustained levels of IFN-γ. (A,B) Representative immunofluorescence images showing kidney tissue at week 1 and 3 post injection, immunostained for nephrin and APOL1 (Abcam) (A), and WT1 and APOL1 (Proteintech). (B). Asterisks indicate glomeruli without staining. Scale bars: 20 µM. (C) Quantification of WT1-stained cells in glomeruli of mice (as indicated) 1 and 3 weeks after injection. Each symbol represents one mouse (*n*=3 mice per genotype at each time point). Counts are the average number of WT1-positive cells in 35 glomeruli. **P<0.005, ****P<0.0001. At 3 weeks, G1/G1 and G2/G2 are significantly different from G0/G0, G1/G1 and G2/G2 mice at 1 week, and from G0/G0 at 3 weeks.

Podocyte injury and loss is thought to be an important factor in the pathogenesis of FSGS and other APOL1 nephropathies (Campbell and Tumlin, 2018; Lim et al., 2016). To determine whether there is evidence of this in *APOL1*-transgenic mice, immunofluorescence was used to evaluate expression of the podocyte markers nephrin and WT1 over time. In G1/G1 and G2/G2 mice, nephrin expression appeared fragmented and staining intensity was reduced 1 week post injection compared with that of G0/G0 mice. Glomerular nephrin expression was no longer evident in some G1/G1 and most G2/G2 mouse glomeruli at 3 weeks post injection (Fig. 7A). These changes in nephrin, an important component of the slit diaphragm, suggest serious podocyte damage (Martin and Jones, 2018; Li et al., 2015; Verma et al., 2018). Similar to nephrin, segmental and global loss of WT1 was apparent 3 weeks post injection, with more global loss seen in G2/G2 mice (Fig. 7B). Because WT1 is a podocyte transcription factor, loss of WT1 usually indicates podocyte loss due to cell death, detachment or de-differentiation (Lefebvre et al., 2015; Liu et al., 2020; Macconi et al., 2006). These results are consistent with the idea that APOL1-induced podocyte toxicity is mediating the development of FSGS (Campbell and Tumlin, 2018; Beckerman et al., 2017).

### APOL1 risk variant toxicity is dependent on the amount expressed

**Fig. 8.**
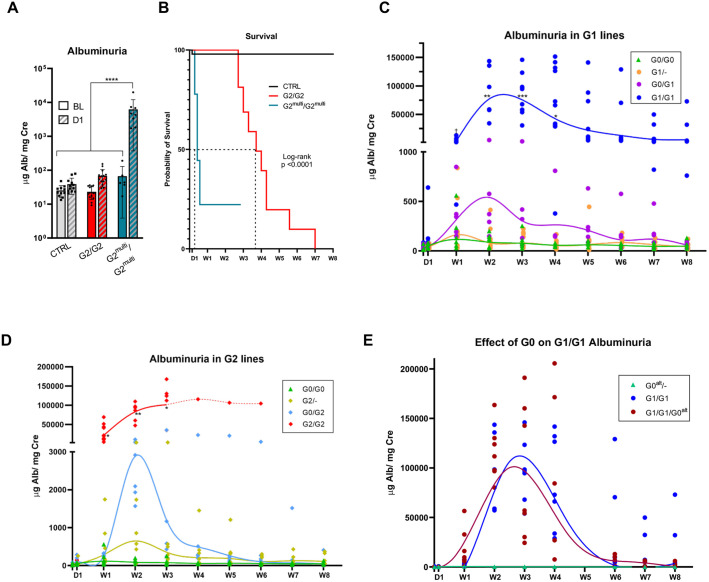
**APOL1 protein levels and zygosity influence toxicity; the presence of the APOL1 allele G0 does not provide protection from G1 toxicity.** (A,B) Comparison of G2^multi^/G2^multi^ and G2/G2 mice were compared regarding proteinurea (A) and survival (B). Quantification of the urinary albumin (Alb) to creatinine (Cre) ratio in mice before (BL) and 1 day after (D1) injection with pCpG-Muγ (A). Plotted are the mean values ±s.d. with individual data points. ****P<0.0001, two-way ANOVA with Tukey's multiple comparisons test comparing all means. (B) Plotted is the rate of survival for individual mouse lines as indicated in weekly increments up to week 8 (W8) post injection. CTRL, FVB control. Log-rank test *P* value <0.0001. A subset of the G2/G2 data are also shown in Fig. 3. (C,D) Albuminuria in G1 (C) and G2 (D) heterozygous (G0/G1 or G0/G2) and hemizygous (G1/− or G2/−) mice, with homozygous (G0/G0, G1/G1 or G2/G2) and control FVB mice (CTRL) for comparison. Individual data points are shown with splines modeling the data trends. ^†^*P*<0.05 is different from G0/G0 and hemizygous mice; *P<0.05, **P<0.01, ***P<0.001, compared to G0/G0, hemizygous, and heterozygous mice; mixed-effects model with Tukey's multiple comparisons test. No differences were seen in heterozygous mice containing the G0 allele comprising both S342G and I384M versus those containing G0^I384M^, i.e. comprising only the I384M part of this allele (data not shown). (E) Albuminuria in G1/G1 and G1/G1/G0^alt^ mice; the Alb:Cre ratio for G0^alt^/− mice is shown as a reference. No significant differences were observed between G1/G1 and G1/G1/G0^alt^ at any time point; mixed-effect model with Tukey's multiple comparisons test. Individual data points are plotted with a smoothing spline to visualize the trend over time. Baseline measurements are shown on the *y*-axis.

Previous studies have suggested that the toxicity of APOL1 risk variants depends on its expression, with increasing protein levels causing a more-severe phenotype (Beckerman et al., 2017; Datta et al., 2020). To better understand the importance of APOL1 protein expression in this model system, the phenotype of G2^multi^/G2^multi^ mice was compared to that of G2/G2 mice. Western blot analysis confirmed that levels of APOL1 were ∼tenfold higher in G2^multi^/G2^multi^ mice following pCpG-Muγ injection (Fig. S12A). At 1 day post injection, levels of urine albumin were unchanged in G2/G2 mice, but levels in G2^multi^/G2^multi^ mice were significantly increase (∼100-fold above baseline) (Fig. 8A). Furthermore, the median rate of survival for G2^multi^/G2^multi^ mice was 3 days, indicating a more-toxic phenotype than that observed in G2/G2 mice (Fig. 8B). These results suggest an increased toxicity with more high risk APOL1 expression.

One persistent question regarding APOL1-mediated toxicity and its relevance to human disease is why two risk variants are required for what appears to be a gain-of-function effect of the variant proteins (Bruggeman et al., 2019). One hypothesis for recessive risk inheritance is that the presence of a G0 allele is protective against the toxicity caused by G1 or G2 (Limou et al., 2015). To address this issue, hemizygous and heterozygous mice were generated by crossing homozygous G0/G0, G1/G1 or G2/G2 lines with control mice (FVB) or with each other. Hemizygous mice have one copy of *APOL1* (indicated as G1/−, G2−). qPCR confirmed that hemizygous mice with one *APOL1* allele expressed half as much APOL1 as the homozygous and heterozygous mice with two *APOL1* alleles (Fig. S12B). Albuminuria was measured weekly and compared in G1 and G2 hemizygous, heterozygous and homozygous mice (Fig. 8C,D). For both G1 and G2 hemizygous mice, there was no difference in albuminuria compared with that in G0/G0 mice. These results are consistent with a recessive mode of inheritance and suggest that, whereas the amount of APOL1 is important, there is not a linear relationship between APOL1 expression and disease severity. As previously suggested, there may be a minimum amount of APOL1 expression required for the development of any toxicity (Bruggeman et al., 2019; Chun et al., 2019; Datta et al., 2020).

Mice heterozygous for both risk variants also had significantly less albuminuria compared with homozygous mice, but trended towards having more albuminuria compared with hemizygous mice. The observation that G0/G2 mice and G0/G1 mice show a trend towards more albuminuria than G2/− and G1/− mice suggests that presence of G0 does not reduce G1 or G2 associated toxicity, at least in a manner that can be observed in this system.

To determine whether G0 has a protective effect when added to an overtly toxic genotype, G1/G1 homozygous mice were crossed with the G0^alt^/− mice, producing mice with one copy of G0^alt^ and two copies of G1 (G1/G1/G0^alt^). An allelic discrimination assay confirmed that these mice expressed both G1 and G0 (Fig. S12C). Albuminuria was measured over 8 weeks in both G1/G1/G0^alt^ mice and G1/G1 mice post treatment with IFN-γ. No significant differences were seen (Fig. 8E). Furthermore, no significant differences regarding histology or survival were seen between mice of these genotypes (Figs S11D, S13). Overall, the results from these modified transgenic lines suggest that the recessive nature of APOL1-nephropathy risk in this model is not easily explained by the G0-mediated rescue of risk variant toxicity.

## DISCUSSION

Chronic kidney disease (CKD) is a large public health burden that disproportionately impacts African Americans and other individuals of recent sub-Saharan African ancestry, in part owing to coding variants in the *APOL1* gene (Murphy et al., 2016; McClellan et al., 2006; Umeukeje and Young, 2019). APOL1-nephropathy, driven by relatively common and penetrant variants, provides an opportunity to understand and more effectively treat a significant subset of patients with kidney disease. Despite a decade of research, it is unclear how APOL1 risk variants cause kidney disease. In part, this is due to the lack of an animal model that faithfully recapitulates human disease and complicated by the fact that non-primates do not express endogenous APOL1. In this study, we describe a mouse model of APOL1-nephropathy that not only resembles human disease in its response to a known disease trigger but also addresses questions regarding mode of inheritance and toxicity. We show that *APOL1* BAC transgenic mice homozygous for the risk variants G1 and G2, but not G0, develop overt kidney disease in response to IFN-γ, which is characterized by high albuminuria, increased serum creatinine and BUN, glomerulosclerosis and podocyte loss, and eventual death. Furthermore, we show that the S342G G1 mutation, one of the two variants that – together – define G1, is necessary for the development of disease. We observe that, in this model, G1 and G2 differ in terms of severity. Last, we demonstrate that the severity of disease correlates with the level of APOL1 expression and cast doubt on the hypothesis that G0 is protective against risk-variant toxicity.

The development of mouse models for APOL1-nephropathy has been challenging owing to the fact that *APOL1* is not present in mice. Previous mouse models have shown the development of FSGS-like disease, albeit in a cDNA overexpression system that lacks regulatory regions and is confounded by supraphysiological levels and GFP tags (Beckerman et al., 2017; Kumar et al., 2018; Kumar et al., 2019). Whereas another APOL1-G1 BAC (C57BL/6 background) mouse model developed proteinuria following injection with recombinant IFN-γ, it did not develop FSGS-like disease, probably due to the transient increase in APOL1 expression (Aghajan et al., 2019). Thus, the field lacks an APOL1 mouse model in which APOL1 expression is controlled by its endogenous regulatory elements, and can be activated in a robust and sustained fashion to induce disease.

To develop a mouse model that mirrors human biology, we utilized a BAC that contains human *APOL1* with its native regulatory sequences and confirmed that a single copy of the BAC integrated into a non-coding region of the FVB genome. Our original injections yielded one mouse with multiple BAC copies and one mouse that would not breed to produce homozygous mice, highlighting the importance of controlling for integration site and expression levels. G0 and G2 mice were generated using CRISPR mutagenesis in the G1 mice, so that the integration site and copy number remained the same across the mouse lines used for direct comparisons. We also paid careful attention to the *APOL1* haplotypes of these transgenes. Because of the reference haplotype listed as ‘reference’ at the NCBI website, we speculate that many experiments that have examined *APOL1* in cell or animal models have utilized G1 and G2 on a reference background (228M and 255R). However, the G1 and G2 variants are not observed on this background in the human population. A recent study suggests these haplotype differences influence toxicity (Lannon et al., 2019). The BAC clone utilized in this study contains a lysine (K) at 150, consistent with the common G0 haplotype but inconsistent with G1 and G2. Therefore, we used CRISPR to mutate aa 150 to ensure that the G1 and G2 alleles were on haplotypes that contain the same coding sequence as exists in humans (Thomson et al., 2014).

These transgenic mice expressed APOL1 in similar amounts and tissues as humans and, like humans, showed no kidney phenotype at baseline. This finding, observed in other *APOL1* BAC models, is consistent with the notion that a second hit is needed to cause disease (Okamoto et al., 2018; Aghajan et al., 2019). Continuous treatment with interferon, a potent stimulator of APOL1 expression, has been shown to induce FSGS in individuals with high-risk *APOL1* genotypes (Markowitz et al., 2010; Nichols et al., 2015). Like in humans, continuous interferon exposure (via injection of an IFN-γ-expressing plasmid) in mice with high-risk *APOL1* genotypes results in albuminuria, elevated serum creatinine, podocyte foot process effacement and lesions that strongly resemble FSGS in humans. IFN-γ, which upregulates levels of APOL1 *in vitro*, increased APOL1 protein levels in all our transgenic mouse lines. Previously published mouse models of APOL1 overexpression demonstrate disease even in the low-risk genotype. In contrast, our G0/G0 mice do not develop disease, which supports the idea that APOL1 is not expressed at supraphysiologically toxic levels in this model (Datta et al., 2020; Kovacsics and Raper, 2014; O'Toole et al., 2018).

Although we demonstrated that mouse IFN-γ leads to binding of IRF1 to the *APOL1* promoter, it is worth noting that mouse and human IFN-γ differ in specificity, which could have influenced the phenotype observed by us (Aguet et al., 1988; Mestas and Hughes, 2004). It is also possible that strain-specific immune responses and susceptibility to kidney damage cause this phenotype to be altered in other mouse strains (e.g. C57BL/6J), although major changes are unlikely, given the large differences between G1/G2 and G0 (Rabe and Schaefer, 2016; Sellers, 2017; Sellers et al., 2012; Szade et al., 2015). Furthermore, whereas the upregulation of APOL1 seems central to its toxicity, it remains possible that other interferon induced molecules, i.e. eIF2α, APOL3 and the NLRP3 inflammasome, are also involved in APOL1-associated disease pathogenesis (Okamoto et al., 2018; Uzureau et al., 2020; Beckerman et al., 2017).

Although APOL1 is highly expressed in the circulation, there is strong evidence that it is the expression of risk variant APOL1 in the kidney that is responsible for the development of CKD (Beckerman et al., 2017; Aghajan et al., 2019; Bruggeman et al., 2014; Reeves-Daniel et al., 2011; Freedman et al., 2016; Freedman et al., 2015). Within the kidney, pCpG-Muγ treatment induced expression of *APOL1* mRNA and APOL1 protein synthesis primarily in podocytes, supporting the idea that podocytes are crucial in at least some APOL1-nephropathies (Beckerman et al., 2017). Injury to podocytes, the epithelial cells that form a key element of the filtration barrier with interdigitating foot processes, is central to the collapsing FSGS variant seen after treatment with interferon (Markowitz et al., 2010). Although APOL1 does not appear to be expressed in foot processes, podocytes in both G1 and G2 mice showed fragmentation of the slit diaphragm protein nephrin and foot process effacement just 1 week after treatment, coinciding with the onset of proteinuria. Because podocytes are terminally differentiated cells, they are particularly susceptible to damage and loss, properties thought to be driving factors in FSGS (D'Agati, 2008; Campbell and Tumlin, 2018). We observed loss of the transcription factor WT1, indicating podocyte loss, by 3 weeks post injection. How the risk variants cause podocyte injury and whether loss is due to podocyte death, detachment or dedifferentiation remains to be determined.

In addition to modeling important features of human APOL1-nephropathy, this coisogenic *APOL1* model provides an opportunity to examine genotype differences. Although the two G1 mutations almost always occur together, there are instances of S342G and, less frequently, I384M occurring alone (Limou et al., 2014; Kasembeli et al., 2015). By using a mouse line that contained only the I384M G1 mutation, we demonstrated that the S342G mutation is necessary for the development of kidney disease. Our results support previous studies that have suggested that the I384M mutation is not required for G1 toxicity or trypanosome lysis (Kopp et al., 2011; Thomson et al., 2014). Whereas both G1 mutations are located on the C-terminus in the serum resistance–associated (SRA)-interacting domain (AA339-398), the I384M mutation is closest to the G2 deletion (388-389), whereas the S342G mutation is located within a different putative α-helix (Sharma et al., 2016). Future studies will be needed to determine whether, despite their physical distance, the G2 variant and the G1 S342G variant cause toxicity through the same mechanism (i.e. protein misfolding). There are differences between G1 and G2 SRA binding as well as protection against trypanosome subspecies, suggesting that the two variants function differently at least in the context of trypanosome lysis (Genovese et al., 2010; Thomson et al., 2014; Cooper et al., 2017). In this model, G2/G2 mice develop a more-severe phenotype than G1/G1 mice, raising the possibility that there are differences in toxicity between the two risk variants. It is worth noting that the frequency of G2 was higher (present in 7/7 genotyped patients) in the published case series with interferon-induced FSGS compared with other forms of non-diabetic CKD (Nichols et al., 2015; Markowitz et al., 2010). Thus, it is possible that the G1/G2 differences observed in this model apply more to interferon-induced disease than other presentations of APOL1-nephropathy.

Increasing evidence, including toxicity in transgenic mouse models, supports the idea that G1 and G2 are toxic gain-of-function mutations (Shah et al., 2019; Beckerman et al., 2017; Johnstone et al., 2012; Olabisi et al., 2016). However, recessive gain-of-function mutations are rare, which has led to the hypothesis that the G0 allele is protective against risk variant toxicity in the G0/G1 or G0/G2 heterozygous state (Limou et al., 2015). Despite many experiments investigating how APOL1 risk variants cause toxicity, the recessive mode of inheritance is not well understood. In part, this is because many studies have utilized overexpression models, which make it difficult to address the contribution of different alleles. We determined that a single copy of a risk allele is insufficient to induce kidney disease in our model, and that the addition of a *APOL1* G0 allele (heterozygosity) – if anything – increased proteinuria, but still to a level that is markedly reduced compared with risk variant homozygotes. Therefore, the recessive nature of disease – i.e. why is disease not seen in the heterozygous state if the risk variant forms are toxic? – is not easily explained by a ‘rescue’ effect of G0 on G1 or G2. Although some *in vitro* studies are consistent with a protective effect of G0, many have demonstrated the importance of APOL1 quantity and even suggested that G0 can be toxic when overexpressed (Beckerman et al., 2017; Datta et al., 2020; O'Toole et al., 2018). Our results showing that hemizygous mice do not develop disease, combined with the fact that the G2^multi^/G2^multi^ phenotype was more severe than the G2/G2 mouse, supports this idea that the amount of risk variant protein is a central factor in driving toxicity. Why an apparent threshold exists and how it can be modified by different environmental factors remains unclear. Future studies utilizing this model will aim to address these questions.

Although APOL1-nephropathy risk primarily fits a recessive model, there is a small increased risk of FSGS, HIVAN and H-ESKD in G1 heterozygotes (Kasembeli et al., 2015; Kopp et al., 2011; Genovese et al., 2010). Although not significant in the given sample sizes in our study, there was a trend towards increased proteinuria in the heterozygous mice compared to control and hemizygous mice, i.e. ∼5× more proteinuria in heterozygotes compared with that in hemizygotes. It is possible that the risk for heterozygotes is context dependent and that the results in this study are specific to IFN-γ-induced FSGS. Our results, combined with previous mouse and human data, suggest that the presence of *APOL1* G0 may actually increase risk variant toxicity in certain circumstances.

Because no toxicity was observed in the G1 or G2 hemizygous mice, our heterozygous mice did not fully address whether *APOL1* G0 may offer protection when coexpressed with a toxic species. To investigate this idea further, we crossed risk-variant mice showing toxicity (G1/G1) with mice that harbor *APOL1* G0 at a different genomic insertion site. No protection was seen with the addition of this G0 allele with regards to proteinuria, histology and survival (Fig. 8E, Figs S11D, S13), although it is possible that subtle differences in disease trajectory may be observed with a larger sample size. Although interpretations are limited by the fact that only one *APOL1* G0 allele was added to the G1/G1 genotype, our results suggest that G0 is not protective against risk-variant toxicity. This finding has important implications for therapeutic development.

Whereas injection of an *IFN-γ*-expressing plasmid allowed us to achieve sustained IFN-γ protein levels in this model, it is limited by its uncontrolled expression and the fact that it requires hydrodynamic injection. Hydrodynamic injection functions by forming pores in the plasma membrane, primarily in the liver. Although our analysis of liver enzymes suggested that liver damage did not mediate any of the observed phenotypes, the effects of liver damage on APOL1-toxicity cannot be ruled out completely. Hydrodynamic injection also causes an early spike in serum IFN-γ to levels that exceed those seen in mice or humans with viral infections. These high IFN-γ levels do not seem to have any adverse effects on control FVB mice; however, it is possible they are influencing the phenotype in a way that is physiologically irrelevant. Other methods of continuous interferon delivery, such as viral vectors or inducible transgenic mice, will be worth exploring in future studies. It will also be of interest to see how these same mice respond to other known kidney stressors, such as viral infections or reduced nephron mass. Because the odds ratio for human APOL1 disease risk varies depending on disease manifestation, finding out how these mice respond to different triggers may help us better understand the mechanism of APOL1 toxicity.

In summary, we have described a mouse model that expresses human *APOL1* together with its normal regulatory sequence and develops FSGS-like disease in response to IFN-γ, a known inducer of FSGS in *APOL1* high-risk patients. We believe this to be the first model to demonstrate the development of reproducible and consistent FSGS lesions in response to a known inducer of human disease (Aghajan et al., 2019; Beckerman et al., 2017; Kumar et al., 2019). Because these transgenic mice model many different features of APOL1-nephropathies, i.e. proteinuria, sclerosis, kidney dysfunction, they will be useful for both identifying mechanisms of injury and testing therapeutic interventions at different stages of disease.

## MATERIALS AND METHODS

### Ethical statement

All practices involving animals were performed in compliance with an animal protocol approved by the Institutional Animal Care and Use Committee at Beth Israel Deaconess Medical Center (BIDMC).

### Generation of the BAC mouse model

#### Coisogenic G0, G1 and G2 transgenic mice

*APOL1* transgenic mice were generated using a bacterial artificial chromosome (BAC) containing a human library clone (CTD2333M18) inside a pBeloBac11 vector (BACPAC Resource Center, Children's Hospital Oakland Research Institute). The 100,104 bp clone contained the *APOL1* gene as well as ∼25 kB of upstream (including exons 1-5 of the *APOL2* gene) and ∼65 kB of downstream (including exons 4-41 of the *MYH9* gene) sequence. The *APOL1* genotype of this *APOL1* BAC was determined to be G0. Next, a G1 BAC was created using site-directed mutagenesis (S342G and I384M for G1; Yale O'Brien Center Transgenic Core Facility). This G1 BAC was introduced into fertilized FVB/NJ oocytes by pronuclear injection (BIDMC Transgenic Core Facility) and founders were selected.

As it had been derived from a G0 BAC comprising p.E150K, the original APOL1 G1 BAC, has a K residue at aa position 150 (Thomson et al., 2014). To create a mouse with the same G1 coding sequence haplotype that exists in humans (Lannon et al., 2019), CRISPR mutagenesis was performed on the G1 pronuclear stage zygotes to change the K at aa position 150 to E, generating the native G1 line (BIDMC Transgenic Core). The BAC integration site for this mouse was determined by DNA sequencing to be in a non-coding region within chromosome 4 (chr4:3789915-37905148) by using whole-genome sequencing (Cergentis, Utrecht, The Netherlands). This mouse line was then used to generate a congenic G2 BAC transgenic line after two rounds of CRISPR, reverting the G1 mutations to the wild type sequence and deleting aa 388-389. An additional mouse line was generated from the G1 mouse by using CRISPR to yield a G342S substitution, leaving the second G1-defining SNP substitution (G0^I384M^ line). A second round of CRISPR was then used to change the M residue at position 384 to I, generating a congenic G0 mouse. Genotypes were confirmed by DNA sequencing. Guide RNAs used for CRISPR are listed in Table S1. Potential off-target cut sites were identified by using Cas-OFFinder (Bae et al., 2014) and verified to be unaltered by using PCR (Table S1). Homozygous and heterozygous mice, i.e. G0/G0, G1/G1, G2/G2, G0/G1 and G0/G2, were generated by crossing mouse lines.

#### Additional transgenic mice

An additional G2 BAC was created using site-directed mutagenesis (deletion of aa 388-389 for G2; Yale O'Brien Center Transgenic Core Facility). This BAC and the original G0 BAC were introduced into fertilized FVB/NJ oocytes by pronuclear injection (BIDMC Transgenic Core Facility) and founders were selected. The resulting G0 line (G0^alt^) was unable to breed to homozygosity and, subsequently, was only used for the G0 rescue experiments. The G2 line (G2^multi^) contained multiple copies of BAC in tandem. G1/G1/G0^alt^ mice were generated by crossing G1 with G0^alt^ mice, the latter having a different integration site. The mouse lines used are summarized in Fig. 1B and Table S2.

### Mouse husbandry and breeding

Transgenic *APOL1* mice were housed in static micro isolator cages (Allentown, Inc.), at a non-barrier conventional animal facility (Beth Israel Deaconess Medical Center ARF). Mice were housed at a maximum of five littermates per cage, with *ad libitum* food and H_2_0. All strains were maintained on a standard 12 h light cycle, at room temperature and humidity (20-22°C and 30-70%, respectively) as per the Guide for Care and Use of Laboratory Animals.

### Generation and injection of the pCpG-Muγ plasmid

The pCpG-Muγ plasmid was generated as previously described (Mitsui et al., 2009). Briefly, the BglII/NheI mouse IFN-γ cDNA fragment (Integrated DNA Technologies, Coralville, IA) was inserted into the BglII/NheI restriction sites of the pCpGfree-mcs vector (InvivoGen, San Diego, CA). Transformation was performed using *E. coli* GT115 cells, and Zeocin™-resistant clones were selected and sequenced using the primer pair 5′-CTGTCTATGCCTGGGAAAGG-3′ (forward) and 5′-CAAACCACAACTAGAATGCAGTG-3′ (reverse). IFN-γ inserted into the CpG dinucleotide-free vector pCpGfree-mcs (pCpG-Muγ) and pCpGfree-mcs alone (pCpG-EV) were grown in *E. coli* GT115 cells and purified using the EndoFree Plasmid Maxi Kit (Qiagen, Hilden, Germany). Both plasmids were administered by hydrodynamic tail vein injection as previously described (Mitsui et al., 2009; Kovacsics and Raper, 2014) to yield sustained serum IFN-γ levels (pCpG-Muγ) or as control (pCpG-EV). Briefly, for each mouse 0.3 µg of plasmid solution was diluted in 2 ml of sterile 0.9% NaCl solution (Injection USP; McKesson Medical-Surgical, Irving, TX, USA) and injected within 5 s using a 1/2 inch 27 gauge needle. Mice were monitored on heating pads until fully recovered.

### Albuminuria quantification

Dilution factors for the albumin ELISA were determined by SDS-PAGE using 5 μl urine and a BSA standard; staining was with PageBlue™ (Thermo Fisher Scientific, Waltham, MA, USA). Urinary albumin and creatinine levels were determined by using the Mouse Albumin ELISA (E99-134, Bethyl Laboratories, Montgomery, TX, USA) and Creatinine Companion (1012, Ethos Biosciences, Philadelphia, PA, USA) kits according to the manufacturers' protocols. Albuminuria was computed as a ratio of urinary albumin to creatinine.

### Animal sacrifice and tissue harvest

Upon completion of each experiment, mice were perfused (by cardiac puncture) for glomerular isolation or tissue harvest. Mice were initially anesthetized in an induction chamber under ≤5% isoflurane and then moved to a surgical field where anesthesia was maintained through inhalation of 1.5-3% isoflurane in O_2_ via a nose cone. For general tissue harvest, mice were perfused with Hank's balanced salt solution (HBSS), and kidney and liver were removed. Each kidney was bisected and halves were prepared for western blotting, histology and electron microscopy. Glomerular isolation was performed as previously reported (Takemoto et al., 2002). Briefly, mice were perfused with inactivated Dynabeads^®^ (14013, Thermo Fisher Scientific) in HBSS and kidneys were removed and minced. Tissue was digested using collagenase A (1 mg/ml) for 45 min, under rotation at 37° C. Digested tissue was passed through two 100 µM strainers, washed with HBSS and spun at 200 g for 5 min at 4°C. Glomeruli were separated from tubules using a magnet and purity was confirmed under the microscope.

### Western blot analysis

Following isolation, glomeruli and kidney halves designated for western blotting were frozen and stored at −80° C. Samples were thawed on ice and lysed in RIPA buffer (Boston Bioproducts, Ashland, MA, USA) containing protease and phosphatase inhibitors (cat. nos: 05892970001, 04693116001, respectively; Millipore Sigma, Burlington, MA, USA). Kidney halves were homogenized with the Tissue Master 125 (Omni International, Kennesaw, GA, USA). Protein concentrations were determined using DC protein assay (Bio-Rad, Hercules, CA, USA) and equal amounts of protein in sample buffer were loaded onto Criterion™ TGX™ Precast Gels (Bio-Rad). Protein was transferred onto PVDF membrane (Bio-Rad) which was blocked in 5% non-fat milk in TBST and incubated with primary (overnight at 4°C) and secondary (1 h at room temperature) antibodies. Antibodies were rabbit anti-APOL1 (Abcam, ab252218) 1:1000, rabbit anti-APOL1 (Sigma, HPA018885) 1:1000, rabbit anti-pSTAT (Cell Signaling, 58D6) 1:500, sheep anti-transferrin (Abcam, ab9033) 1:5000, goat anti-nephrin (R&D, AF3159) 1:500, mouse anti-vinculin (Sigma, V9131), and GAPDH-HRP (Genetex, GT239) 1:5000. Blots were detected using Pierce™ ECL Western Blotting Substrate (Thermo Fisher Scientific) and imaged on film or FluorChem E Imager (Proteinsimple, San Jose, CA, USA). Antibody information is provided in Table S3. APOL1 antibodies were confirmed to be specific by using control mice (Fig. S1).

### RNA isolation and analysis

RNA was isolated from glomeruli using the AllPrep DNA/RNA/Protein Mini Kit (Qiagen). cDNA was synthesized from 100 ng of RNA using the High Capacity cDNA Reverse Transcription Kit (4368814, Thermo Fisher Scientific). Multiplexed qPCR was performed on a QuantStudio 6 Flex Real-Time PCR System with TaqMan Gene Expression Master Mix (4370048) and gene expression assays (APOL1: Hs01066280_m1; IRF1: Mm01288580_m1; GAPDH: Mm99999915_g1; Thermo Fisher Scientific). Relative quantification was performed using the delta-delta Ct method. Allelic discrimination assay was performed as previously described, but with cDNA instead of gDNA (Ruchi et al., 2015). Briefly, PCR was performed on 10 ng cDNA using *APOL1*-specific primers (F- 5′-GCCAATCTCAGCTGAAAGCG–3′; R: 5′-TGCCAGGCATATCTCTCCTGG-3′). Purified PCR product (Cat. no. 28106, Qiagen) was digested using HindIII-HF or NspI (R3104 and R0603, New England Biolabs) and run on a 2% agarose gel.

### Plasma and serum analyses

Blood samples were collected through tail vein sampling or cardiac puncture prior to perfusion. Each cardiac puncture sample was divided for serum collection by centrifugation, plasma collection was carried out using Lithium heparin collection tubes (365985, BD Biosciences, Franklin Lakes, NJ, USA). Owing to the small sample sizes, tail vein samples were reserved solely for serum isolation. All samples were spun at 2000 g, at 4°C for 15 min, after which the supernatant was collected, and aliquots taken and frozen at −80C.

Serum IFN-γ levels were quantified using a commercially available mouse IFN-γ ELISA kit (50-173-19, Invitrogen, Carlsbad, CA, USA) according to the manufacturer's instructions. Serum sample aliquots were sent to the BIDMC Small Animal Imaging Facility (LSAIF) for potentiometric quantification of BUN, ALT, and AST (Vitros 350 Chemistry System, Ortho Clinical Diagnostics). Serum creatinine quantification was performed using isotope dilution liquid chromatography - tandem mass spectrometry (LC-MS/MS) at the UAB/UCSD O'Brien Center Core C. For western blotting, plasma was diluted 1:10 in 1× protease and phosphatase inhibitors in H_2_0, and SDS-PAGE was carried out as described above.

### Histology

Once harvested, tissue samples were fixed in 10% formalin for 24 h, and then washed and stored in PBS. Fixed tissue was paraffin embedded, sectioned at 5 μm thickness and stained using Periodic acid–Schiff (PAS) or Mason's trichrome stain (BIDMC Histology Core). PAS and trichrome slides were imaged using a BX60 compound fluorescent microscope and DP73 camera (Olympus, Tokyo, Japan).

### Electron microscopy

For transmission electron microscopy evaluation (TEM), mouse kidney tissues were fixed by immersion in 2.5% glutaraldehyde and 2% paraformaldehyde in 0.1 M cacodylate buffer pH 7.4 (modified Karnovsky's fixative), post-stained in 1% osmium tetroxide, dehydrated and embedded in Epon for thin sectioning. Grids were imaged on a JEOL 1400 TEM.

### Immunofluorescence

Unstained paraffin-embedded sections (described above) were deparaffinized in xylene, followed by washing in graded ethanol dilution. Slides were washed twice with double-distilled H_2_0, unmasked with IHC-tek Epitope Retrieval Solution (IHC World, Woodstock, MD, USA) in a steamer for 45 min, and cooled to room temperature. Sections were washed 3× with 1× PBS and incubated in blocking solution (2% BSA, 0.3% Triton X-100 and 2% goat serum) for 1 h at room temperature. Slides were incubated overnight with primary antibody at 4°C, washed 3× with PBS, and incubated in secondary antibody for 1 h at room temperature. Sections were washed 4× with PBS, incubated in 10 µg/ml Hoechst (1:1000 in 1×PBS) for 5 min, and mounted with ProLong™ Gold (Thermo Fisher Scientific). Immunofluorescence was imaged using a Zeiss LSM 880 upright laser scanning confocal microscope and Zeiss Imaging software (Zeiss, Oberkochen, Germany). Co-staining of APOL1 with calnexin and nephrin was imaged using a Zeiss Confocal system with airy scan processing as previously described (Chun et al., 2019). Airy scan processing was performed using ZEN Black (Zeiss) software with default 2D airy scan processing parameter settings. SIM analysis of kidney section samples, stained for APOL1, endomucin and nephrin, was performed using a Zeiss Elyra SP.1 system, as described previously (PMID: 30733285; 31924668). The 34-micron-period grating was shifted 5× and rotated 5×. SIM processing of acquired images was performed using Zeiss ZEN software with default processing parameter settings. See Table S4 for antibody information. APOL1 antibodies were confirmed to be specific by using control mice and IgG controls (Fig. S1).

### *In situ* hybridization

*In situ* hybridization was performed at the Harvard Medical School Neurobiology Imaging Facility on formalin-fixed, paraffin-embedded kidney sections using Advanced Cell Diagnostics (ACD, Newark, CA, USA) protocols. *APOL1* mRNA was detected using the RNAScope^®^ Multiplex V2 Assay (ACD) with the probe Hs-APOL1-No-XMm (Cat. no. #459791); positive and negative controls were run in parallel. Following *in situ* hybridization, sections were co-stained with FITC-conjugated wheat germ agglutinin (Sigma-Aldrich, L4895-2MG, 1:100) to identify glomeruli and tubule structures.

### Podocyte cell lines and chromatin immunoprecipitation

Glomeruli were harvested from non-injected mice as described above and cultured in RPMI-1640, 10% heat-inactivated FBS, insulin-transferrin-selenium supplement (Sigma-Aldrich), and penicillin-streptomycin (Thermo Fisher Scientific). After 3 days of culture and podocyte outgrowth to at least 50% confluence, cells were immortalized using a lentivirus expressing the temperature-sensitive SV40 large T tsA58 antigen (Applied Biological Materials, Richmond, BC, USA). Cells were transduced with the SV40 lentivirus in the presence of polybrene for 4 days and then were passaged and cultured in selection media (growth medium with 2 µg/ml puromycin, Invivogen). For qPCR and chromatin immunoprecipitation (ChIP) experiments, G0/G0 podocytes were cultured at 37°C (growth restrictive) for 4 days and then treated with 10 ng/ml recombinant murine IFN-γ (PeproTech, Rocky Hill, NJ) for 6 h. Cells were fixed in 1% formaldehyde for 10 min and ChIP was performed using the ChIP-IT Express kit (Active Motif, Carlsbad, California) with an antibody to IRF1 (D5E4, Cell Signaling Technology) and Rabbit IgG (2729S, Cell Signaling Technology). Eluted ChIP DNA was purified using the Chromatin IP DNA Purification Kit (Active Motif) and real time PCR was run using PowerUp™ SYBR™ Green Master Mix (Thermo Fisher Scientific) and primers spanning the *APOL1* transcription start site (F: 5′-AGCTGCTGGGAAGTTGTGAC-3′ and R: 5′-ATCCCACCTCCAGTTATGCG-3′).

### Necropsy

Mice were cut open and submerged in 10% formalin for 6 h and then sent to the Harvard Medical School Rodent Histopathology Core for necropsy. Several organs (heart, lungs, pancreas, spleen, liver, kidneys, stomach, intestines and cecum, brain and spinal cord, reproductive organs, and bladder) were sectioned, stained and examined by the core.

### Statistical analysis

Statistical analyses were performed using the GraphPad Prism 8.0 software (La Jolla, CA, USA). Baseline albuminuria was compared using a one-way ANOVA and time-course albuminuria was compared using the mixed-effects repeated-measures model. Differences in serum BUN, creatinine and liver enzyme markers (ALT/AST) were assessed using a two-way ANOVA. Tukey's multiple comparison test was used for post-hoc comparisons, generating multiplicity adjusted *P*-values. Survival curves were compared through the Log Rank test, with Bonferroni's correction to account for multiple comparison bias. WT1-positive cell counts were compared using two-way ANOVA with Tukey's multiple comparisons test comparing every mean. All data are presented as means±s.d. unless otherwise indicated. *P*<0.05 was considered significant.

## Supplementary Material

Supplementary information
