# Supplementary material for: Recessive, gain-of-function toxicity in an APOL1 BAC transgenic mouse model mirrors human APOL1 kidney disease
Source: Dis Model Mech. 2021 Aug 5;14(8):dmm048952. doi: 10.1242/dmm.048952 (PMC8353097; doi:10.1242/dmm.048952)
Supplement: Supplementary information [file dmm-14-048952-s1.pdf]

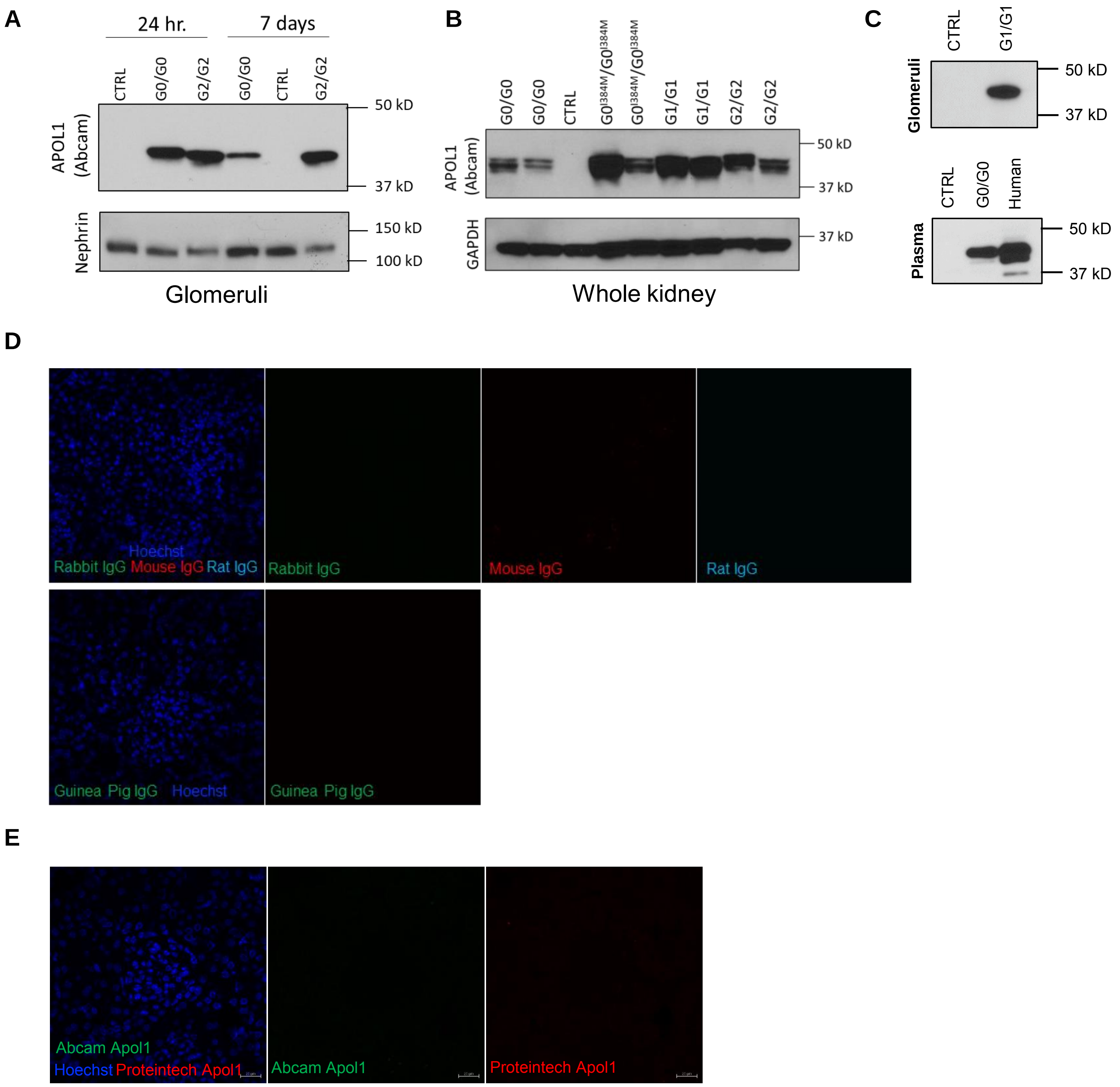

Fig. S1. Antibody specificity

The specificity of antibodies for western blot was verified using control FVB mice that do not contain the *APOL1* BAC. (A) Blot showing the Abcam APOL1 antibody in glomeruli from mice 24 hours or 1 week after pCpG-Muγ injection, Nephrin is shown as a loading control. (B) Blot showing the Abcam APOL1 antibody in whole kidney from untreated mice, GAPDH is shown as a loading control. (C) Blot showing the Sigma APOL1 antibody in mouse glomeruli 2-days post-injection and in untreated mouse and human plasma. The specificity of antibodies for immunofluorescence staining was verified using both IgG controls and by using control FVB mice. (D) Immunofluorescence antibody controls using rabbit, mouse, rat, and guinea pig IgG with the corresponding secondary antibodies. (E) Immunofluorescence staining of APOL1 (Abcam and Proteintech antibodies) in a control FVB mouse, 1 day after pCpG-Muγ injection.

Disease Models & Mechanisms • Supplementary information

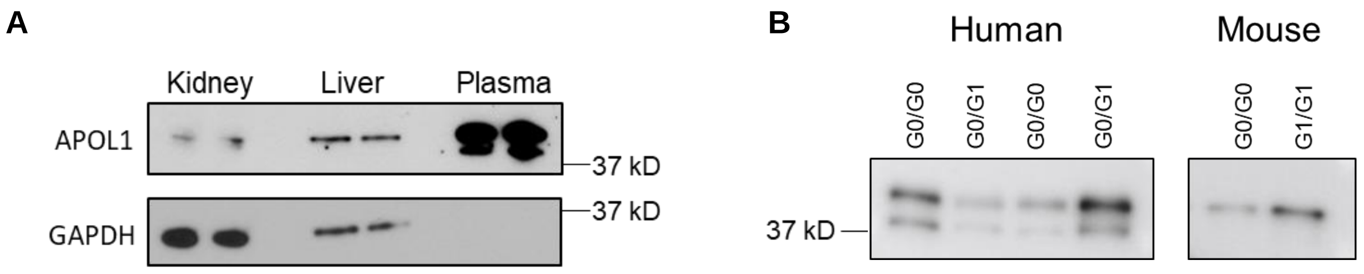

Fig. S2. APOL1 expression in uninduced transgenic mouse lines

APOL1 expression in the untreated transgenic mouse lines was measured by western blot. (A) APOL1 (Abcam) expression in the kidney (50 µg lysate), liver (20 µg lysate), and plasma (5 µg lysate) isolated from the same $G0^{I384M}$ mice 1 day post-injection; n =2. GAPDH is shown as a loading control for kidney and liver lysates. (B) APOL1 (Sigma) expression in 20 ug of plasma from 4 different humans and 2 different mice (untreated), genotypes indicated above the sample.

Disease Models & Mechanisms • Supplementary information

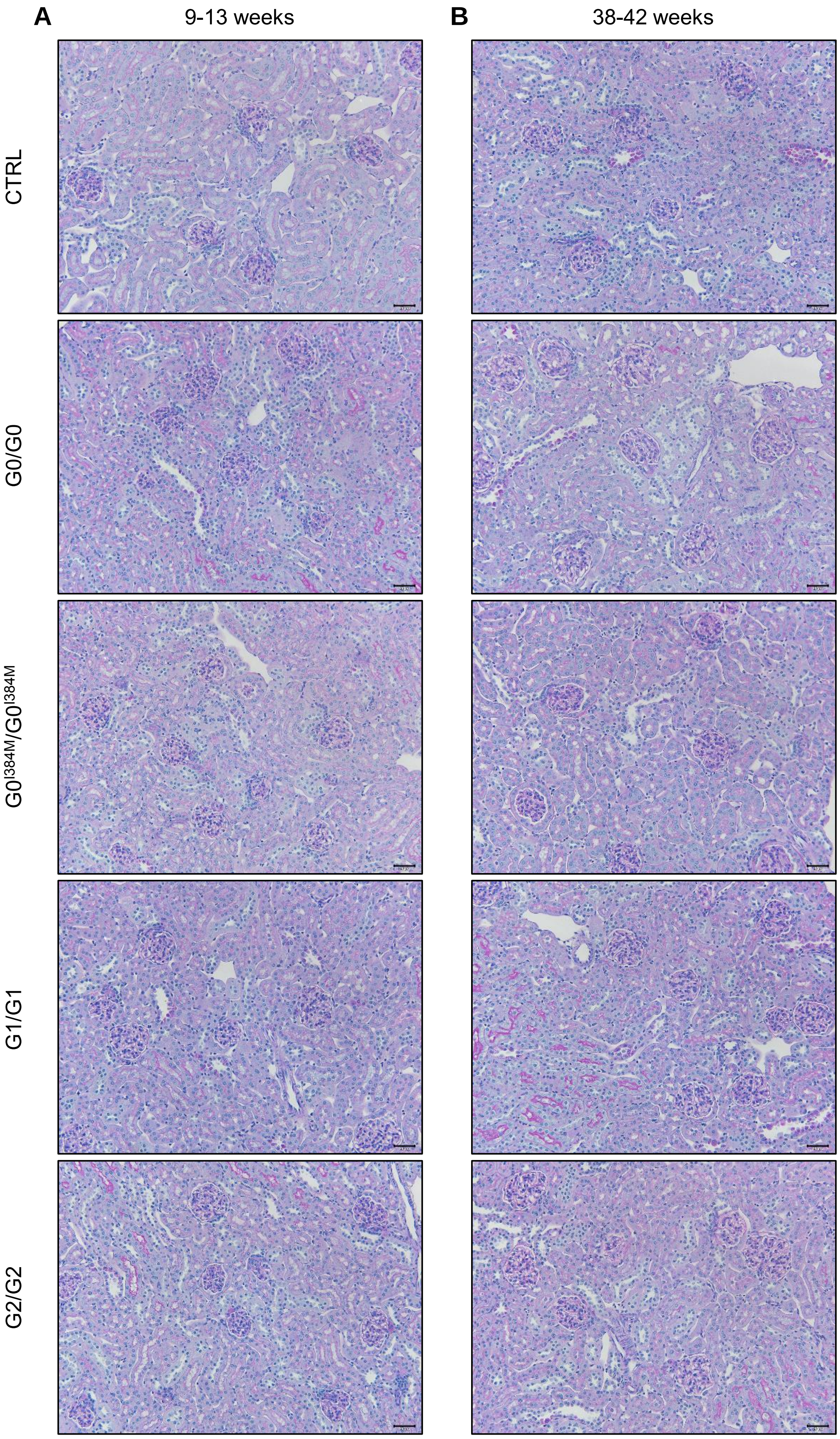

Fig. S3. Untreated APOL1- transgenic mice show no histological kidney damage
Representative PAS images from the kidney cortex of control and APOL1-transgenic mice at 9-13 (A) and 38-42 (B) weeks old. Scale bar is 20 microns. No morphological differences were observed

Disease Models & Mechanisms • Supplementary information

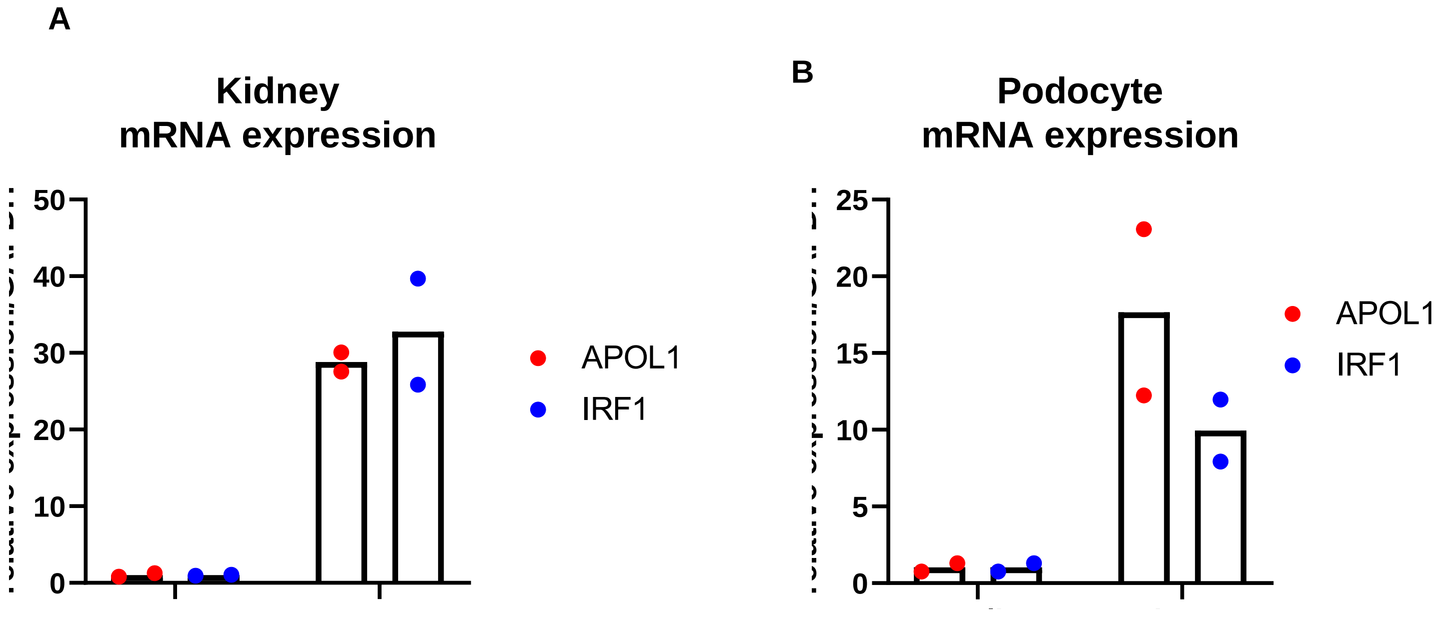

Fig. S4. IFN-γ effect on mRNA levels

mRNA expression of APOL1 and IRF1 in (A) G2/G2 mouse kidney at baseline or 24-hours after pCpG-Muγ injection and (B) G0/G0 mouse podocytes 6-hours after IFN treatment. Expression is normalized to Gapdh, n= 2 per time point.

Disease Models & Mechanisms • Supplementary information

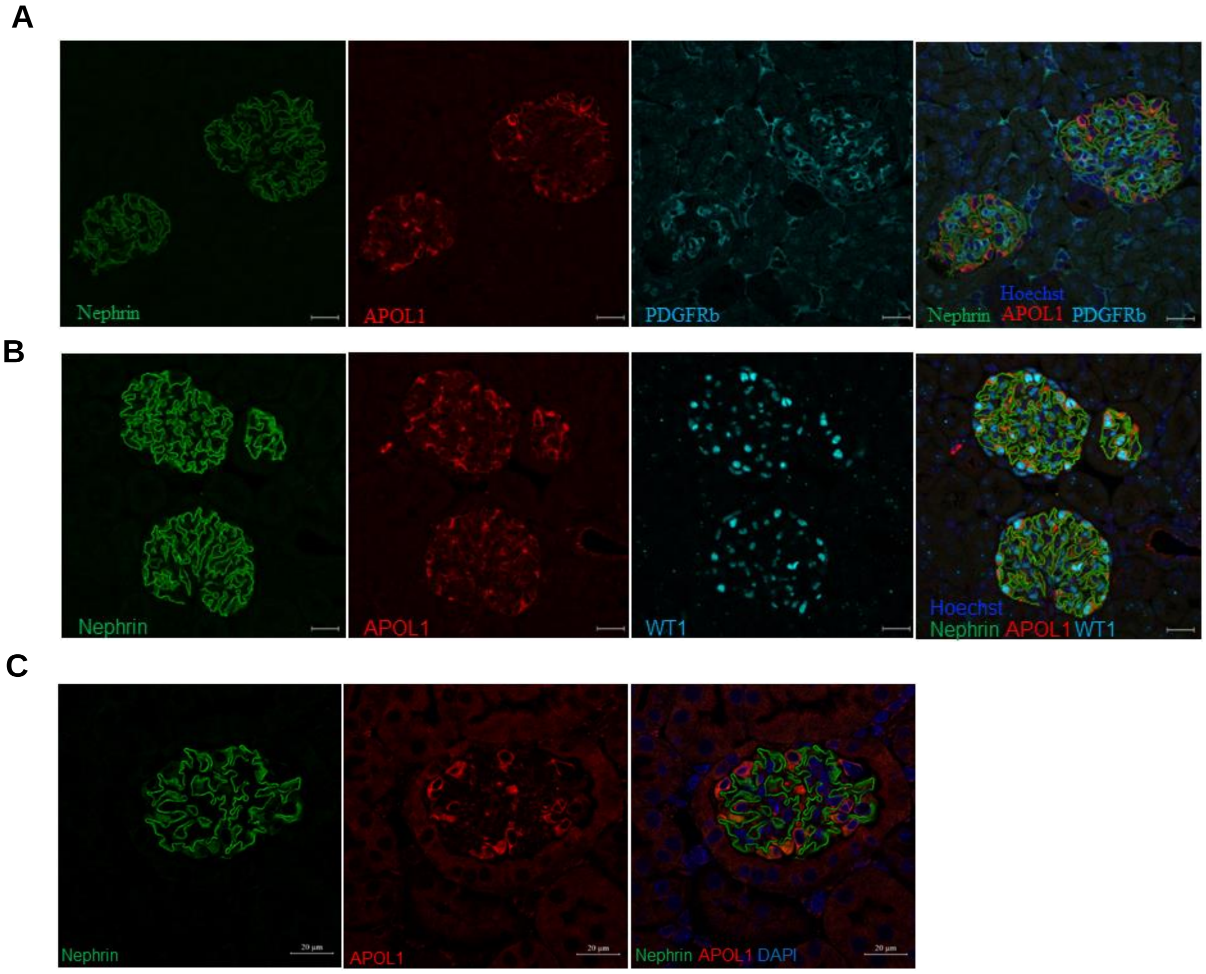

Fig. S5. APOL1 is induced in podocytes following pCpG-Muγ injection

Immunohistochemistry in kidney from G1 mice 24 hours after plasmid injection. Co-staining with Nephrin, APOL1 (Proteintech), and PDGFRb (A) or WT1 (B). Scale bar = 20 μM. (C) Co-staining with Nephrin and APOL1 (Sigma). APOL1 cell-type localization did not differ by APOL1 genotype.

Disease Models & Mechanisms • Supplementary information

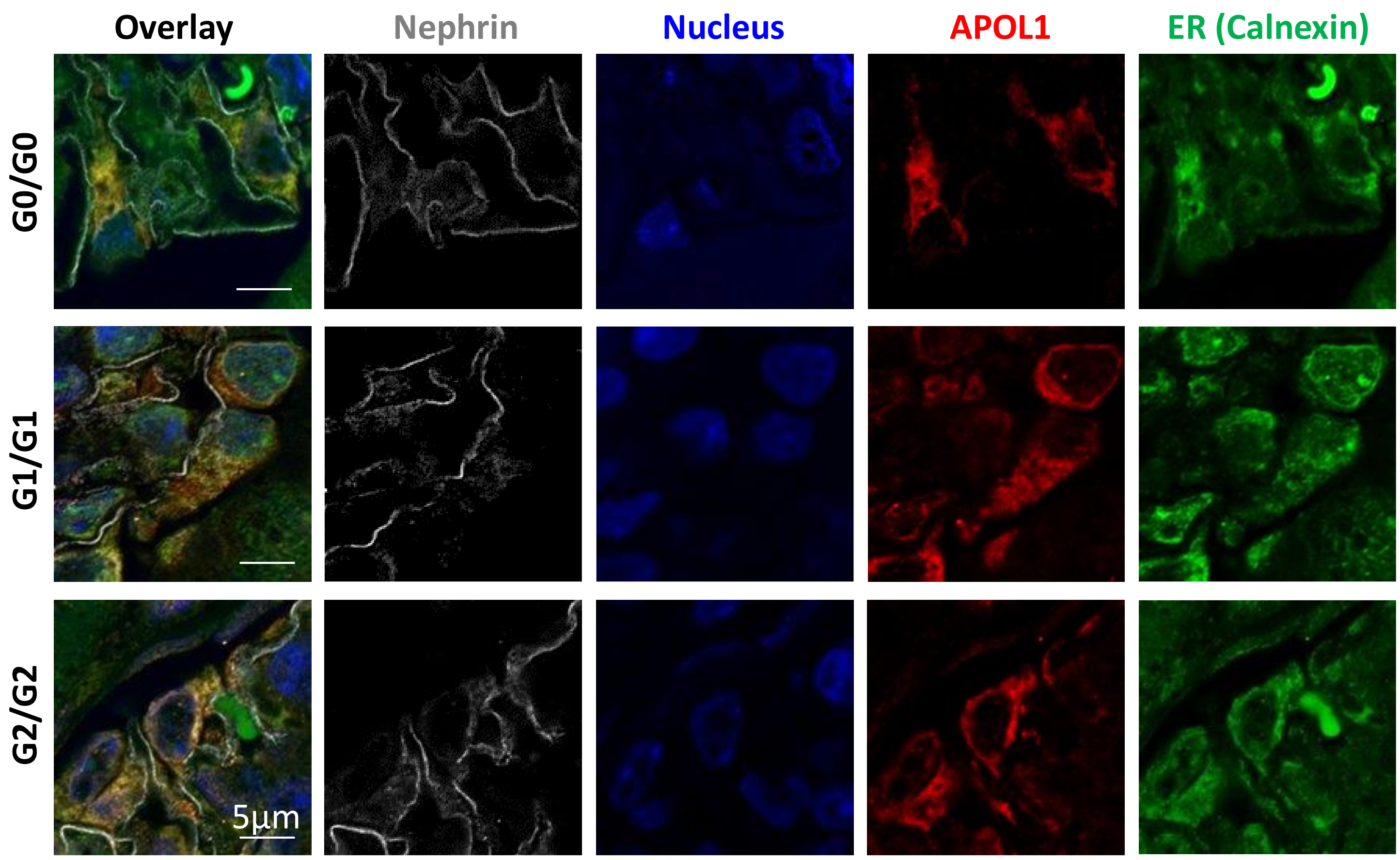

Fig. S6. APOL1 co-localization with the Endoplasmic Reticulum (ER)

Kidney sections from G0/G0, G1/G1 and G2/G2 mice 2 days post-injection co-stained with APOL1 (Abcam 1:50), the ER marker Calnexin (1:50) and Nephrin (1:200). The overlay shows some co-localization between APOL1 and the ER. Nephrin is shown to outline the podocytes. Image acquisitions were performed using a Zeiss Confocal system with airy scan processing as described previously (Chun et al., PNAS 2019). Airy scan processing was performed using ZEN Black (Zeiss) software with default 2D airy scan processing parameter settings.

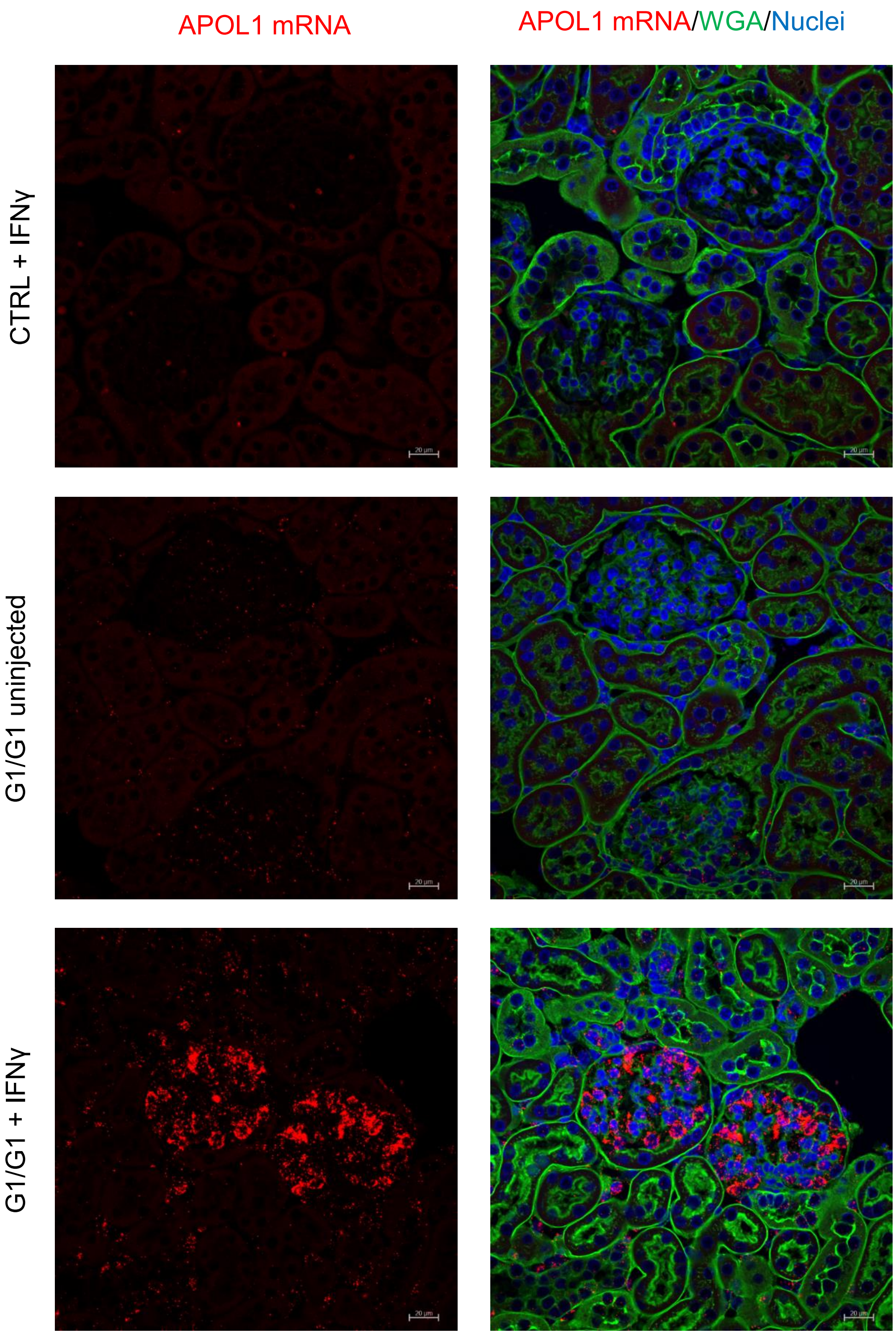

Fig. S7. Apol1 mRNA is localized within the glomeruli

Apol1 *in situ* hybridization on kidney from Control and G1/G1 mice either uninjected or injected with pCpG-Muγ (24 hours). Tissue was co-stained with the lectin WGA (green) and Hoescht (blue). RNA is primarily localized within the glomeruli of injected G1/G1 mice, but there is some weaker staining in the tubules.

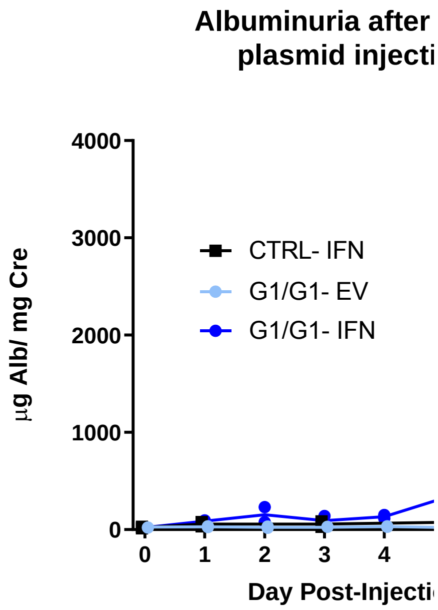

Fig. S8. Albuminuria after pCpG plasmid injection

Albumin to creatinine ratios following pCpG-Muγ (IFN) or pCpG-EV (EV) injection in control or G1/G1 mice. Day 0 shows baseline levels pre-injection. N = 2 per group, individual data points shown with line connecting the means.

Disease Models & Mechanisms • Supplementary information

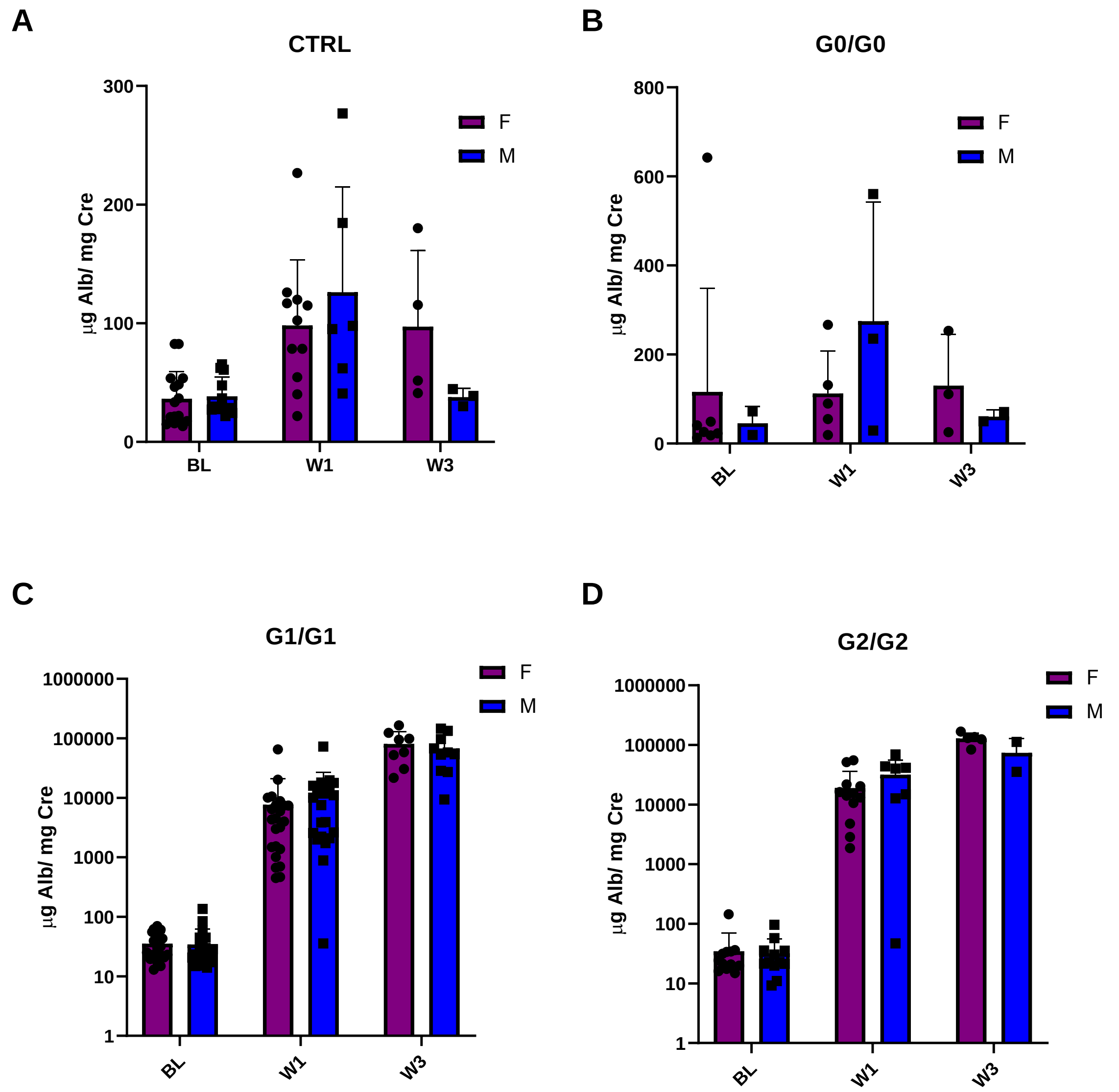

Fig. S9. Albuminuria in APOL1 transgenic mice is not sex dependent

Albumin to creatinine ratio for control (A), G0/G0 (B), G1/G1 (C), and G2/G2 (D) mice following injection with pCpG-Muγ plasmid. Individual data points from several experiments are shown at baseline (BL), week 1 (W1) and week 3 (W3). No significant differences were found using a one-way ANOVA with Sidak's multiple correction's test.

Disease Models & Mechanisms • Supplementary information

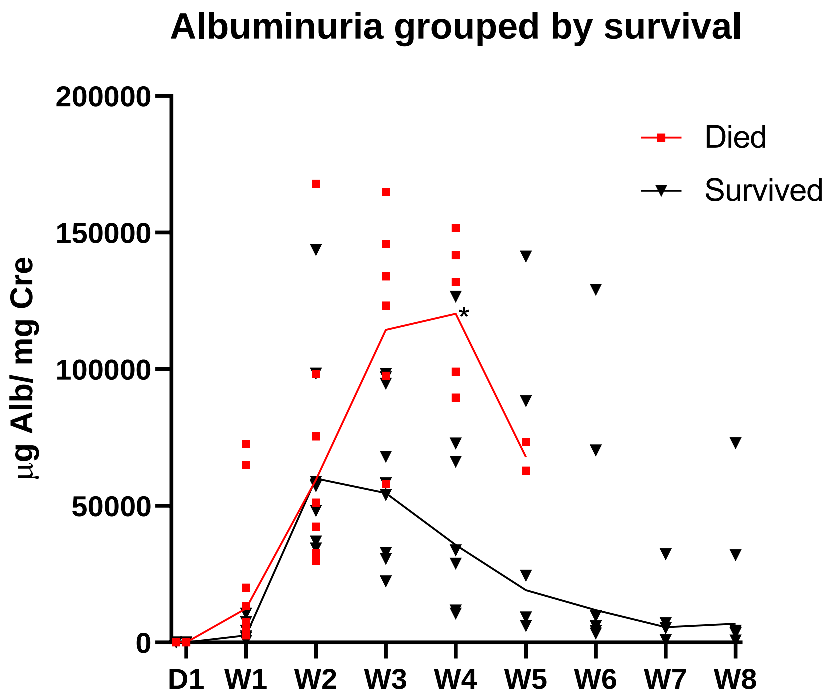

Fig. S10. Difference in proteinuria between G1 mice that survived and those that died.

Albuminuria over time for G1 mice that either died before of survived until 8-weeks post-injection. Individual data points are shown with a line connecting the geometric means. * p value < 0.05, mixed-effects analysis with Sidak's multiple comparisons test.

Disease Models & Mechanisms • Supplementary information

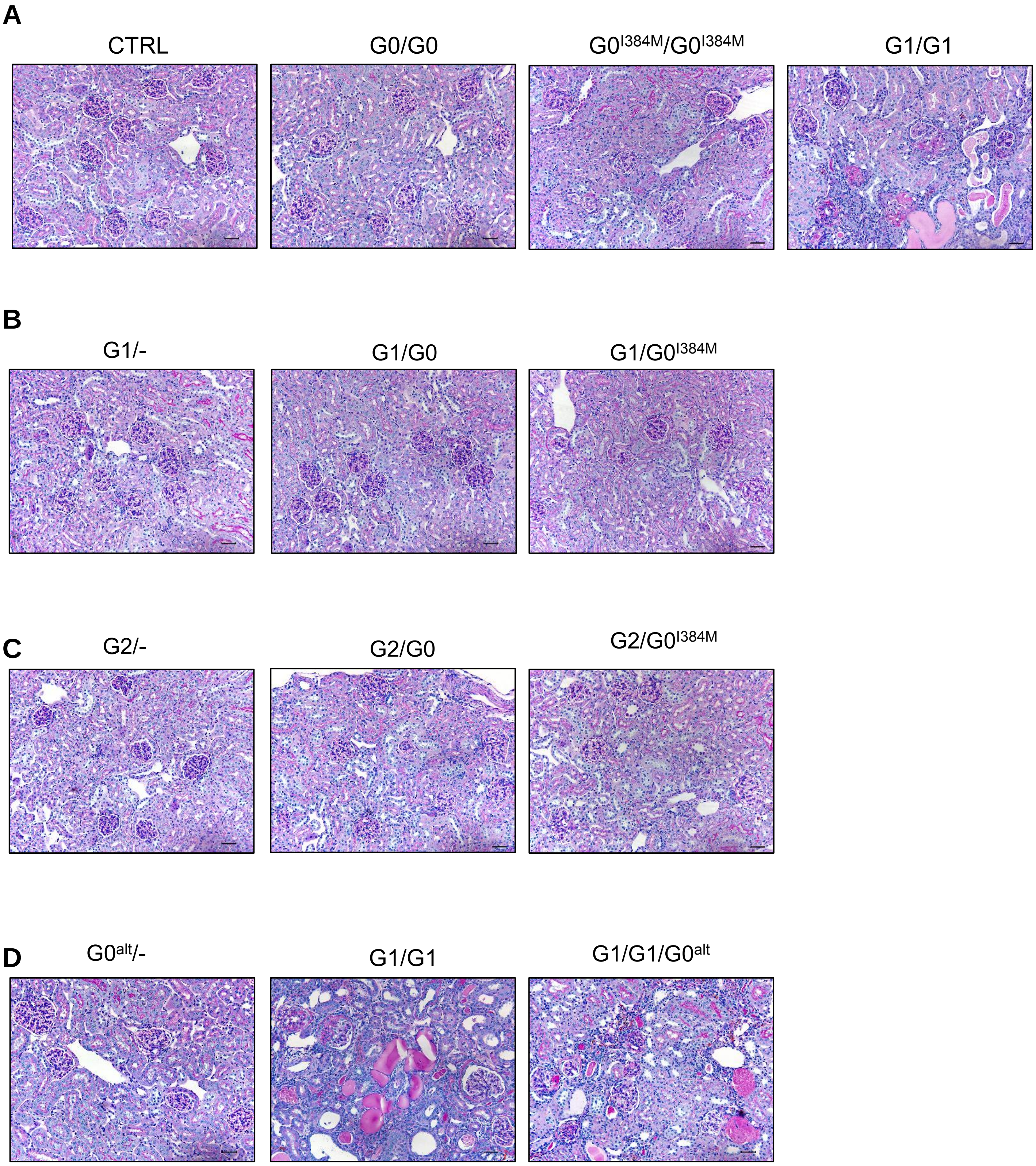

Fig. S11. PAS images 8-weeks post-injection

Representative PAS kidney images 8-weeks post-injection from (A) CTRL, G0/G0, $G0^{I384M}/G0^{I384M}$, and G1/G1 mice. (B) G1 and (C) G2 hemizygous and heterozygous mice. (D) $G0^{alt}$, G1/G1, and G1/G1/ $G0^{alt}$ mice. Scale bar = 20 μM

Disease Models & Mechanisms • Supplementary information

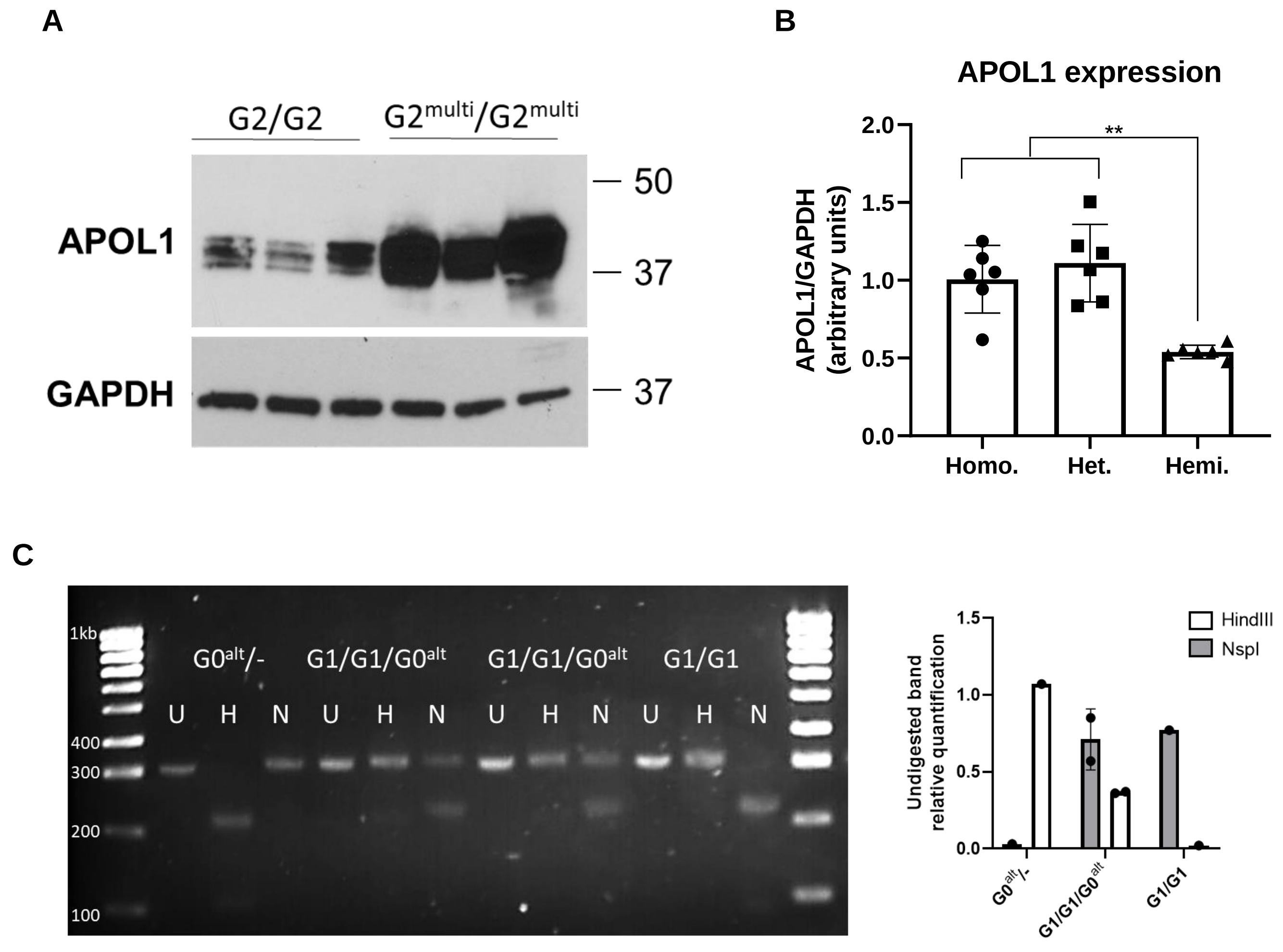

Fig. S12. APOL1 protein expression

(A) Representative western blot showing APOL1 expression in kidney from G2/G2 and G2^{multi}/G2^{multi} mice 24 hours after injection with pCpG-Muγ, n=3 per group. GAPDH is shown as a loading control. (B) Relative glomerular APOL1 expression between homozygous, heterozygous and hemizygous mice 24-hours after injection. ** $p < 0.01$, One-way ANOVA with Tukey's multiple comparisons test. (C) Gel from allelic discrimination assay showing undigested product (U), product digested with *HindIII* (H) which only cuts G0, and product digest with *NspI* (N) which only cuts G1. Quantification for the undigested product with each enzyme relative to undigested sample is shown to the right. Glomerular cDNA samples (1-day post-injection) are from two G1/G1/G0^{alt}/- mice, with G0^{alt}/- and G1/G1 mice shown as controls. G1/G1/G0^{alt} mice express ~2X more G1 RNA (undigested with *HindIII*) than G0 RNA (undigested with *NspI*).

Disease Models & Mechanisms • Supplementary information

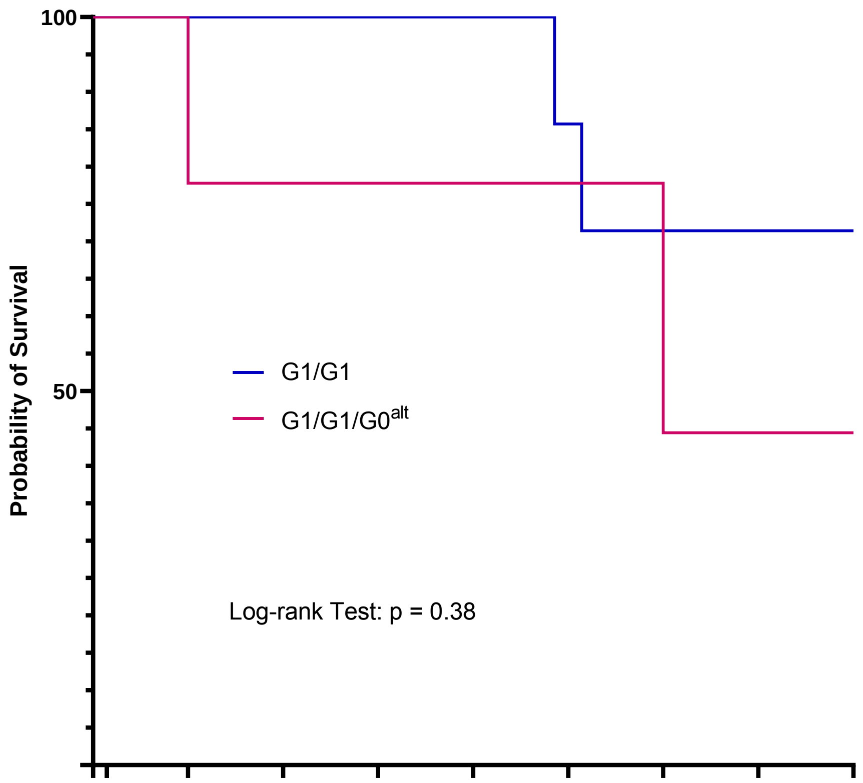

Fig. S13. Survival of G1/G1 and G1/G1/G0alt mice

Survival curves for G1/G1 and G1/G1/G0^{alt} mice over the course of an 8 week experiment. No significant difference in survival was seen (Log-rank test). N= 9 for G1/G1/G0^{alt}; n=7 for G1/G1 . D indicates day, W indicates week post-injection.

Table S1. Guide RNAs used for CRISPR mutagenesis of transgenic mouse lines and potential off-target cut sites

				off-target sites with fewer than 3 mismatches						primers used for PCR/sequencing	
**From**	**To**	**Mutation site(s) changed**	**gRNA(s)**	**Sequence (mismatch in lower case, PAM sequence underlined)**	**Chrom.**	**location**	**strand**	**# of mismatches**	**annotated gene**	**Forward primer**	**Reverse primer**
G1	G2^{S342G}	384/388	TAATTATAAGATTCTGCAGG	TAATTATAtGATgCTGCAGGAGG	chr3	126,000,486	+	2	none	5'- CAGAGAAGCCAACAGTCATACA- 3'	5'- CATCCCAGAGATATCTGAGCATAAG- 3'
G2^{S342G}	G2	342	AGGAGTCAAGCTCACGGATG								
G1	G0^{I384M}	342	TGTAAGCTTCTTTCTTGTGC	TGTAAGCTTCTagCTTGTGCAGG	chr4	131,530,700	-	2	none	5'- GACCCATGCATGACAGACCT- 3'	5'- GTTTGAGCGGGTTTTGTGGG- 3'
			CTGCTCATTTCCAGGATGCT	CaGCTCcTTTCCAGGATGCTGGG	chr6	94,403,336	-	2	none	5'- GGAAGAGGAAGAGGAAGAAGAAG- 3'	5'- GTGGGAAGTGGTACTTGTAGTC- 3'
G0^{I384M}	G0	384/388	TAATTATAAGATTCTGCAGG	TAATTATAtGATgCTGCAGGAGG	chr3	126,000,486	+	2	none	5'- CAGAGAAGCCAACAGTCATACA- 3'	5'- CATCCCAGAGATATCTGAGCATAAG- 3'

Disease Models & Mechanisms • Supplementary information

Table S2. Transgenic mouse nomenclature used

Symbol	Description
CTRL	FVB mouse with no transgene
G0/G0	Homogzyous BAC transgenic mouse with G0 APOL1 integrated into chromosome 4
$G0^{I384M}/G0^{I384M}$	Homozygous BAC transgenic mouse with G0I384M APOL1 integrated into chromosome 4
G1/G1	Homozygous BAC transgenic mouse with G1 APOL1 integrated into chromosome 4
G2/G2	Homozygous BAC transgenic mouse with G2 APOL1 integrated into chromosome 4
G1/-	Hemizygous BAC transgenic mouse with one copy of G1 APOL1
G0/G1	Heterozgyous BAC transgenic mouse with one copy of G0 and one copy of G1
$G0^{I384M}/G1$	Heterozgyous BAC transgenic mouse with one copy of $G0^{I384M}$ and one copy of G1
G2/-	Hemizygous BAC transgenic mouse with one copy of G2 APOL1
G0/G2	Heterozgyous BAC transgenic mouse with one copy of G0 and one copy of G2
$G0^{I384M}/G2$	Heterozgyous BAC transgenic mouse with one copy of $G0^{I384M}$ and one copy of G2
$G2^{multi}/G2^{multi}$	Homozygous BAC transgenic mouse with multiple copies of G2, integration sites unknown
$G0^{alt}$ /-	Hemizygous BAC transgenic mouse with one copy of G0, integration site unknown
$G1/G1/G0^{alt}$	BAC transgenic mouse homozygous for G1 (chromosome 4) with an additional single copy of $G0^{alt}$ (unknown integration site)

Table S3. Antibodies used for western blot

**Primary antibodies**					
**Antibody target**	**Species**	**Manufacturer**	**Catalog number**	**Dilution used**	**diluent**
APOL1	Rabbit	Abcam	ab252218	1:1000	5% NFDM
APOL1	Rabbit	Sigma	HPA018885	1:1000	5% NFDM
PhosphoSTAT1 (Y701)	Rabbit	Cell Signaling	58D6	1:500	5% BSA
Transferrin	Sheep	abcam	ab9033	1:5000	5% NFDM
Nephrin	Goat	R&D	AF3159	1:500	5% NFDM
Vinculin	mouse	sigma	V9131	1:5000	5% NFDM
GAPDH-HRP		Genetex	GT239	1:5000	5% NFDM
**Secondary antibodies**					
**Antibody target**		**Manufacturer**	**Catalog number**	**Dilution used**	**diluent**
Rabbit anti-Goat IgG, HRP		Thermo Scientific	31402	1:5000	5% NFDM
Donkey anti-sheep IgG, HRP		Thermo Scientific	A16041	1:5000	5% NFDM
Anti-Rabbit IgG, HRP		Cell Signaling	7074	1:5000	5% NFDM
Anti-mouse IgG, HRP		Cell Signaling	7076	1:5000	5% NFDM
NFDM- Non-fat dry milk					

Disease Models & Mechanisms • Supplementary information

Table S4. Antibodies used for immunofluorescence

Antibody target	Manufacturer	Catalog number	Dilution used	Secondary Antibody	Secondary Manufacturer	Secondary Catalog number	Secondary Dilution
Wilms Tumor (WT1)	Abcam	ab89901	1:300	Goat anti-rabbit IgG, Alexa Fluor 647	Thermo Fisher	A-21244	1:250
APOL1	Abcam	ab252218	1:500	Goat anti-rabbit IgG, Alexa Fluor 546	Thermo Fisher	A-11035	1:250
APOL1	Proteintech	66124-1-Ig	1:500	Goat anti-mouse IgG, Alexa Fluor 546	Thermo Fisher	A-11030	1:250
APOL1	Sigma	HPA018885-100UL	1:500	Goat anti-rabbit IgG, Alexa Fluor 546	Thermo Fisher	A-11035	1:250
Nephrin	Progen	GP-N2	1:200	Goat anti-Guinea Pig IgG, Alexa Fluor 488	Thermo Fisher	A-11073	1:250
Endomucin	Life Technologies	14-5851-81	1:200	Goat anti-Rat IgG, Alexa Fluor 647	Thermo Fisher	A-21247	1:250
PDGRFb	Cell Signaling	3169	1:200	Goat anti-Rabbit IgG, Alexa Fluor 647	Life Technologies	A-21244	1:250
Calnexin	Cell Signaling	C5C9	1:50	Goat anti-Rabbit IgG, Alexa Fluor 488	Life Technologies	A-11008	1:250
FITC conjugated Lectin from wheat (WGA)	Sigma	L4895	1:100	N/A			
Rabbit IgG	Santa Cruz	sc-2027	1:250	Goat anti-Rabbit IgG, Alexa Fluor 488	Invitrogen	A-11008	1:250
Guinea Pig IgG	Sino Biological	CR4	1:400	Goat anti-Guinea Pig IgG, Alexa Fluor 488	Invitrogen	A-11073	1:250
Rat IgG	Sigma Aldrich	I4131-10MG	1:400	Goat anti-Rat IgG, Alexa Fluor 647	Invitrogen	A-21247	1:250
Mouse IgG	Santa Cruz	sc-2025	1:150	Goat anti-rabbit IgG, Alexa Fluor 546	Invitrogen	A-11035	1:250
